# Surface-enhanced Raman spectroscopy for label-free cancer liquid biopsy: from fundamentals to clinical analysis of biofluid

**DOI:** 10.3389/fchem.2025.1696979

**Published:** 2026-01-12

**Authors:** Ming Chen, Mingjun Zhao, Yue Cai, Qiong Zhang, Zhenzhen Peng, Qiwen Li, Zhibin Wang

**Affiliations:** 1 Department of Nuclear Medicine, The First Hospital of Lanzhou University, Lanzhou, Gansu, China; 2 Department of Bipharmaceutical Sciences, Faculty of Pharmaceutical Sciences, Shenzhen University of Advanced Technology, Shenzhen, China; 3 Center for Cancer Immunotherapy of Institute of Biomedicine and Biotechnology, Shenzhen Institutes of Advanced Technology, Chinese Academy of Sciences, Shenzhen, China

**Keywords:** cancer, clinical biofluids, deep learning, label-free detection, liquid biopsy, SERS

## Abstract

Cancer remains one of the major public health problems due to its high morbidity and mortality globally. Because metastasis is the major cause of cancer death, developing new approaches for early diagnosis is of paramount importance in this context. Surface-enhanced Raman scattering (SERS) has emerged as a cutting-edge analytical technique. SERS features an exceptional sensitivity and specificity, enabling rapid non-destructive detection of trace-level samples. Therefore, SERS technology is widely used across medical disciplines, particularly in cancer diagnosis for early-stage and non-invasive diagnostic evaluation. Using liquid biopsy with rich metabolic information, SERS has facilitated the identification, analysis, and progression monitoring of various cancers. In this review, we systematically summarize recent advances in label-free SERS-based cancer diagnostics. We first outline the fundamental principles of SERS, key substrate fabrication methodologies, and essential spectral analysis techniques. We then highlight the applications of label-free SERS in liquid biopsy using various biofluids, including blood, urine, saliva, and sweat. Finally, we discuss current challenges and future directions in this rapidly evolving field.

## 1 Introduction

Cancer is a life-threatening disease characterized by uncontrolled tumor growth and high lethality, making early and accurate diagnosis a paramount challenge in modern medicine (Attwaters, 2023). The advent of precision medicine has dramatically changed the landscape of cancer treatment, yet current diagnostic approaches remain imperfect (Moutinho, 2023). These methods are primarily categorized into histopathological examination (e.g., tissue biopsy) and imaging-based techniques (e.g., CT, MRI, PET) (Billimoria and Bhatt, 2023; Lu et al., 2021; Pickhardt, 2022). Although tissue biopsy is the gold standard for tumor characterization, its invasiveness, physical discomfort, risk of metastasis, and limitations due to tumor heterogeneity pose significant clinical challenges (Welch and Bergmark, 2024). Therefore, non-destructive approaches capable of early cancer detection are urgently needed.

Liquid biopsy has emerged as a revolutionary, minimally invasive strategy for precision medicine and cancer management, including screening, early diagnosis, and prognostic assessment (Batool et al., 2023). It detects cancer-specific biomarkers such as circulating tumor cells (CTCs), exosomes, miRNA, and proteins (e.g., PSA, CEA, AFP) in biofluids (Li D. et al., 2023; Sofie Van et al., 2024). However, conventional detection techniques like enzyme-linked immunosorbent assay (ELISA), electrochemical immunoassay, and fluorescence immunoassay are often complex, time-consuming, and possess poor multiplexing capabilities, preventing their use in rapid on-site detection (Humaira et al., 2022; Lorena et al., 2023).

Surface-enhanced Raman scattering (SERS) has garnered significant attention as a cutting-edge analytical technique to address these limitations. SERS features exceptional sensitivity (enabling single-molecule detection) and specificity, providing unique molecular “fingerprint” information for rapid, non-destructive analysis of trace-level samples with minimal sample volumes (Choi et al., 2020). The remarkable signal enhancement in SERS originates from two complementary mechanisms: predominant electromagnetic enhancement via plasmonic amplification at nanostructured metal surfaces, and chemical enhancement through molecule-substrate charge transfer (Ding et al., 2017; Langer et al., 2020). SERS-based detection strategies are broadly categorized into labeled approaches (using functionalized nanoparticles with Raman reporters) (Lei et al., 2023) and label-free methods relying on intrinsic molecular vibrations. While labeled SERS offers high specificity, it requires complex sample pretreatment. In contrast, label-free SERS demonstrates particular utility in liquid biopsy by enabling the direct analysis of native biofluids (e.g., blood, urine, saliva) through their intrinsic molecular fingerprints, thereby preserving holistic biochemical information and streamlining the analytical process (Bi et al., 2023).

This review specifically focuses on the label-free approach for several compelling reasons. First, by eliminating the need for complex labeling procedures, label-free SERS significantly simplifies the workflow, reduces analysis time and cost, and minimizes potential alterations to the native state of biomarkers, which is crucial for reflecting the true physiological conditions. Second, and perhaps more importantly, the label-free modality captures the holistic molecular fingerprint of the biofluid. This untargeted strategy is exceptionally powerful for discovering novel, unexpected biomarkers and for providing a comprehensive metabolic profile of the disease state, going beyond the detection of a few pre-selected targets. While the labeled approach excels in specific, high-sensitivity detection of known biomarkers, the label-free approach offers a broader discovery potential that is particularly advantageous in the early stages of cancer, where specific biomarker panels may not be fully defined. Despite its great promise, the label-free SERS approach faces unique challenges, particularly in the interpretation of complex spectral data, creating a knowledge gap that warrants a dedicated and systematic review to guide future research.

The analysis of these intrinsic molecular fingerprints, however, is challenged by the complexity and high dimensionality of the resulting SERS spectra. Recently, Artificial Intelligence (AI) has become indispensable in this context, as it can be fully integrated with SERS technology to interpret complex signals and differentiate previously indistinguishable spectral differences, opening a broad path for big data analysis (Bi et al., 2023; Lussier et al., 2020). The successful integration of SERS and AI over the past 5 years has yielded satisfactory results in the label-free detection of various cancers, including colon, breast, gastric, and lung cancer (Bi et al., 2023; Lussier et al., 2020), underscoring its transformative potential for intelligent cancer diagnosis.

**FIGURE 1:**
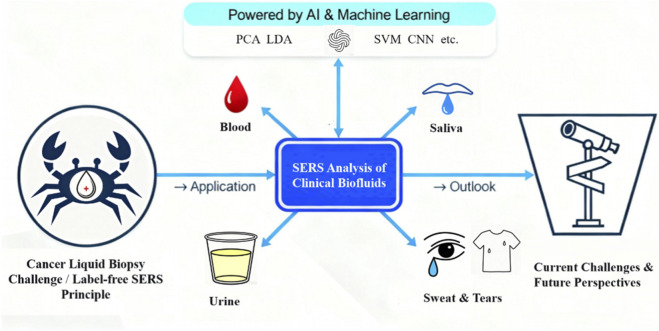
Roadmap of this review: Label-free SERS for cancer liquid biopsy from fundamentals to clinical analysis.

This review is dedicated to the rapidly advancing field of label-free SERS for cancer liquid biopsy and synthesizes recent advances from the past 5 years, with a particular focus on literature published since 2021. Our scope is specifically centered on the application of label-free SERS for cancer detection in liquid biopsy samples, including blood, urine, saliva, sweat, and tears. While numerous excellent reviews cover the broader fundamentals of SERS (Langer et al., 2020) and the application of AI in label-free SERS (Bi et al., 2023), and its growing role in clinical analysis (Li et al., 2025), this work aims to provide a comprehensive update and critical perspective on the translation of this technology to clinical cancer diagnostics. Furthermore, the powerful combination of vibrational spectroscopy and machine learning, as leveraged in this review for cancer diagnostics, is demonstrating its utility across diverse scientific disciplines, including geochemical analysis (Almeida et al., 2025). The logical framework of this review, outlining the journey from fundamental principles to clinical applications and future outlook, is graphically summarized in Figure 1. Accordingly, we systematically highlight key advances, covering: (1) fundamental mechanisms and substrate designs, (2) applications in biofluid analysis (blood, urine, saliva, sweat), and (3) current challenges and future directions. By synthesizing insights across these domains, this review underscores how SERS technology offers a minimally invasive alternative to traditional diagnostic methods, enabling early cancer detection through its unique molecular fingerprinting capability.

## 2 Design and fabrication of SERS substrates

### 2.1 The development of SERS

The development of SERS can be traced back to the Nobel Award discovery of Raman scattering, by Chandrasekhara V. Raman in 1928 (Raman and Krishnan, 1928). The practical application of Raman scattering, however, was fundamentally constrained by its inherently weak signal intensity (approximately one in 10^8^ photons) until the unexpected discovery by Fleischmann’s team, who observed a million-fold signal enhancement from pyridine molecules adsorbed on roughened silver electrodes - a phenomenon later termed SERS (Fleischmann et al., 1974). Initially misinterpreted as merely a surface area effect, this paradigm-shifting observation was subsequently recognized through the seminal work of Jeanmaire, Van Duyne, and Albrecht (1977) as a distinct spectroscopic phenomenon arising from two complementary mechanisms: electromagnetic enhancement via plasmonic amplification at nanostructured metal surfaces and chemical enhancement through molecule-substrate charge transfer (Albrecht and Creighton, 1977; Fleischmann et al., 1974). Over subsequent decades, SERS has evolved into a sophisticated analytical technique with rigorously characterized enhancement mechanisms that now enable remarkable single-molecule detection sensitivity (10^−15^ M) (Han et al., 2022), subcellular-resolution spectral imaging (Wang et al., 2017), and real-time pharmacokinetic monitoring through vibrational fingerprinting (Choi et al., 2020).

Electromagnetic enhancement is believed to exert a predominant influence, evidenced by the molecular insights gleaned from SERS spectra. Its primary mechanism revolves around the localized electromagnetic enhancement field near the metal surface or surface plasmons (Itoh et al., 2017). Upon exposure to external light, metal nanoparticles provoke the excitation of surface plasmons, engendering a potent electric field at the surface. This augmented electric field markedly amplifies the Raman scattering of molecules adsorbed onto the surface. The apex of electric field enhancement occurs when two metal surfaces are in proximity, with a nanometer-scale gap between them, delineating a “hot spot” where the electric field is exceptionally intense. Studies have demonstrated that the enhancement can attain remarkable levels, with an enhancement factor surpassing ∼ 10^10^, originating from the excitation of localized surface plasmon resonance in metallic nanostructures (Yamamoto et al., 2014).

Enhancement factor stands as the predominant metric for assessing the degree of SERS enhancement. There are various methods for estimating enhancement factor, depending on experimental settings and conditions. In essence, enhancement factor quantifies the extent to which Raman signals are amplified in the presence of nanoparticles (SERS condition) relative to the scenario where an equivalent amount of Raman dye exists in the target system sans nanoparticles (native condition). A generalized formula expresses as (I _SERS_/N _SERS_)/(I _bulk_/N _bulk_), where I and N signify the intensity of the Raman signal and the number of molecules (Yoshida et al., 2010).

However, it is crucial to recognize that this standard formula provides a lower-bound, volume-averaged estimate of the enhancement. The reality of SERS is highly heterogeneous. The overwhelming majority of the SERS signal originates from a tiny fraction of molecules adsorbed at nanoscale gaps or sharp tips known as ‘hot spots,’ where the local electromagnetic field is intensely amplified. In contrast, molecules located outside these hot spots contribute negligibly. Therefore, the actual local enhancement factor at an optimal hot spot can be many orders of magnitude higher than the calculated average value. This fundamental aspect of SERS—the extreme spatial localization and non-uniformity of signal generation—is a critical consideration for liquid biopsy applications. It directly impacts the reproducibility and quantitative reliability of measurements, as minor variations in the distribution of analytes relative to hot spots, or in the substrate’s nanostructure itself, can lead to significant signal fluctuations.

The influence of chemical enhancement on the overall signal intensity is generally considered secondary to the electromagnetic mechanism, typically contributing a factor of 10–100 to the total enhancement (Han et al., 2022). It manifests through charge transfer between the analyte molecule and the SERS substrate, which augments the molecular polarizability. A critical aspect of this mechanism is that the chemical interaction modifies the electronic states and the polarizability tensor of the adsorbate. Consequently, the SERS spectrum acquired can exhibit considerable differences compared to the normal Raman spectrum of the free molecule, including shifts in Raman band positions and changes in their relative intensities (Han et al., 2022). This underscores that chemical enhancement not only contributes to the signal intensity but also fundamentally shapes the spectral profile presented for analysis. The efficacy of this mechanism hinges significantly on the specific chemical nature of the analyte and the substrate.

These two enhancement mechanisms do not work in isolation but synergistically to generate the final SERS signal. The electromagnetic mechanism creates the essential precondition by producing an intense local electric field at the “hot spots.” The chemical mechanism then acts upon molecules already within this field, further boosting their Raman scattering efficiency by altering their electron cloud distribution (polarizability). Thus, the overall enhancement is a concerted effect of both processes.

While the electromagnetic contribution dominates the signal amplification in most experimental systems, the role of chemical enhancement becomes significant and even critical under specific conditions. This occurs primarily when two criteria are met simultaneously: (1) the analyte molecule forms a strong chemical interaction with the metal substrate (e.g., gold or silver), such as through adsorption via thiol groups, amine groups, or the π-electrons of aromatic rings; and (2) the energy of the incident laser matches the charge-transfer transition energy at the molecule-metal interface. Under these conditions—exemplified by systems like pyridine on a silver electrode—chemical enhancement not only substantially increases the signal intensity but also profoundly shapes the observed spectral fingerprint (band positions and relative intensities). Consequently, a thorough understanding of chemical enhancement is vital for accurately interpreting label-free SERS spectra, particularly those originating from biomolecules with strong affinities to metal surfaces.

Despite the well-understood theoretical framework, the practical application of these enhancement mechanisms faces significant challenges rooted in their inherent limitations. The electromagnetic mechanism, while capable of enormous enhancement, is critically limited by its extreme sensitivity to the nanoscale geometry of the plasmonic structures. This makes the signal intensity highly vulnerable to minor variations in “hot spot” fabrication, posing a fundamental challenge to reproducibility. In contrast, the chemical mechanism offers better signal uniformity but is limited by its modest enhancement factor (typically 10–100x) and its reliance on specific chemical interactions between the analyte and the metal surface. These intrinsic limitations of both mechanisms underscore a central tenet in SERS: the paramount importance of substrate engineering. It is the precise and uniform design of the substrate (controlling size, shape, and arrangement of nanostructures) that directly dictates the intensity and reliability of the local electromagnetic fields, thereby governing the overall SERS performance and its practical utility.

The profound sensitivity and molecular specificity afforded by these enhancement mechanisms, despite their challenges, have positioned SERS as a powerful technique for biological analysis. It is these very attributes—single-molecule detection capability and rich vibrational fingerprinting—that make SERS exceptionally suited for interrogating complex biological mixtures. This foundational principle directly enables the application at the heart of this review: the analysis of liquid biopsy samples. When coupled with the diagnostic power of artificial intelligence to decipher the complex spectral data, SERS transitions from a physical phenomenon to a transformative tool in clinical oncology.

Altogether, SERS offers several advantages over conventional fluorescence-based techniques, including heightened sensitivity, robust multiplexing capabilities, diminished background interference, and exceptional photostability. Its broad applications span sample identification, analysis, and characterization across diverse fields. Moreover, SERS demonstrates remarkable prowess in detecting biomarkers in bodily fluids such as blood, urine, and saliva, highlighting its significant potential in biomedical sciences (Cardinal et al., 2017; Chao et al., 2022; Joseph et al., 2018; Wang et al., 2017; Wang et al., 2021).

### 2.2 Preparation of SERS substrates

#### 2.2.1 One-dimensional substrates, metal/semiconductor nanoparticles (NPs)

On sensing a one-dimensional substrate, SERS uses nano colloids including silver and gold to mix with the sample to induce the production of biomarkers-NPs. To overcome the limitations of conventional spherical silver and gold NPs, investigators have explored additional metallic nanomaterials with different optimized morphologies. For example, NPs, nanorods, nano stars, nano cubes, nanoflowers, nanocages and nano prisms have been tested (Jin et al., 2023; Paz et al., 2023; Ramos et al., 2018; Yang et al., 2021).Research shows that the dimensions, distribution and morphology of metallic NPs exert a paramount influence on augmenting the SERS signals (Indrajit et al., 2022). Unlike nanospheres, engineered nanostructures exhibit superior SERS enhancement factors, with nanorods particularly notable for their pronounced field enhancement at the tip. Elongating the nanorods extends their longitudinal surface plasmon resonance band into the Near-Infrared spectrum, rendering them conducive for deep tissue biosensing imaging (Li Q. et al., 2023). Nanostructures such as metal nano stars and nano prisms, characterized by higher surface area ratios and single-tip configurations, further bolster reporter gene attachment densities, thereby amplifying the SERS signal (Liebel et al., 2022).

Apart from nanoparticle size and shape, the inter-nanoparticle gaps significantly influence signal amplification by virtue of their role in field enhancement. Close proximity between NPs leads to aggregate formation, concentrating hot spots within the aggregate region and consequently intensifying SERS signals (Qi et al., 2020). However, the drawback of randomly aggregated NPs lies in their inconsistent signal reproducibility, underscoring the importance of precise control over nanoparticle aggregation within the nanometer scale (Son et al., 2022).

One strategy to mitigate aggregate instability involves fabricating NPs with satellite structures, wherein the core is linked to additional plasmonic NPs via organic linkers. This configuration facilitates robust hot spot enhancement through a synergistic interplay between core-satellite and satellite-satellite plasmon coupling mechanisms. To address this issue, the researchers reported a unique SERS sensing platform based on the “core-satellite” structure. To improve the accuracy and stability of the detection, the core-shell NP embedded in the internal standard molecule was synthesized to act as the “core”, and SERS labels were firmly fixed on the prepared core to form a “core-satellite” structure. The characteristic signal is dynamically calibrated by the intensity ratio of the Raman probe to the internal standard molecule due to the smooth construction of this structure. Therefore, stable and strong SERS signal was generated, which improved the sensitivity of the sensing model (She et al., 2021). The “core-satellite” combination has better repeatability than the traditional polymerization system, which promotes the development of SERS sensors.

Cellulose serves as an excellent carrier for metal NPs, enabling the fabrication of highly sensitive and stable SERS substrates. When silver NPs (Ag NPs) are generated *in situ* on cellulose, they form dense SERS “hot spots” within the nanoscale gaps of the cellulose matrix (She et al., 2021).

Further improvements were achieved by using sulfonated cellulose nanofibers (S-CNF), where the abundant hydroxyl and sulfonic acid groups effectively capture silver ions and promote their conversion into well-dispersed Ag NPs. This design ensures strong nanoparticle adhesion and the formation of stable 1D “hot spots.” Notably, the negatively charged S-CNF surface provides electrostatic repulsion, allowing the S-CNF-Ag NP substrate to maintain excellent dispersion stability for over 12 months (Wang W. et al., 2023).

Furthermore, the functionality of 1D colloidal substrates can be enhanced by integration with magnetic beads. In a typical application, functionalized magnetic beads are employed not as the primary SERS-active material, but as a versatile platform for capture, separation, and concentration of target analytes from complex liquid biopsies. This approach contrasts with traditional sandwich-type immunoassays that require cumbersome washing steps. By capturing targets onto the beads and then concentrating them magnetically, the method enables direct SERS detection in the aqueous phase. This strategy offers two key advantages: first, it minimizes potential protein damage during separation; second, promoting analyte aggregation on the bead surface can create additional SERS hotspots when plasmonic nanoparticles are introduced (Lie et al., 2024). Thus, the magnetic beads streamline the assay by eliminating extensive washing while simultaneously concentrating the molecules of interest.

#### 2.2.2 Two-dimensional (2D) substrates

The random dispersion of nanoparticles in conventional colloidal SERS substrates introduces several non-negligible defects, such as an unpredictable distribution of hotspots, heterogeneity in target adsorption, and significant spatial variability, which collectively lead to poor signal stability and reproducibility (Xia et al., 2022; Xu et al., 2023). To overcome these drawbacks of traditional SERS substrates, researchers are exploring diverse avenues, particularly on the development of 2D nanomaterial arrays. These innovative approaches have successfully addressed issues such as poor reproducibility and non-uniform distribution of hotspots, making significant strides in bioanalytical applications (Chen et al., 2020). Typically, 2D solid substrates reliant on metal NPs, and implemented on solid platforms like silicon wafers or slides, are crafted through techniques including *in-situ* reduction and interfacial self-assembly. Their surface plasmon resonance properties offer facile modulation opportunities (Guselnikova et al., 2022; Jin et al., 2022).

Researchers have devised a method to fabricate 2D silver plates by affixing silver NPs onto etched slides using a silver mirror reaction. Concurrently, silver nanoparticle colloids are synthesized as plasmonic enhancers to augment Raman scattering, resulting in the successful detection of characteristic fingerprint peaks from the analyte solution under robust SERS signals. Meanwhile, an independent study engineered a flexible SERS substrate featuring a multi-scale cavity structure adorned with Ni_3_S_2_-modified polyvinylidene fluoride microcavities on nanocavities. The dense 2D nanosheets afford ample surface area for the deposition of plasmonic Ag NPs, thereby enhancing the density and intensity of the “hotspots” (Xu et al., 2024). Further improvements include 2D substrate with self-calibration capability using interfacial self-assembly method. The adaptive surface has a high affinity recognition site for the target, ensuring high specificity and stability of the aptamer sensor. For quantitative analysis, SERS signals generated by reporter genes were calibrated in real time using secondary peaks of silicon. Due to the inherently ordered structure, the nanopore arrays show excellent reproducibility (Yin et al., 2022).

In related work, a self-assembled nanocomposite film was created by combining g-C_3_N_4_ nanosheets with AuNPs, forming a dual-functional platform for photocatalytic degradation of organic dyes and SERS monitoring. The g-C_3_N_4_/AuNPs nanocomposite features an organized structure of g-C_3_N_4_ thin films and a monolayer of closely packed AuNPs, fabricated via liquid-liquid interface self-assembly. This design promotes uniform distribution of hotspots for reliable SERS detection while providing active sites for photocatalytic reactions (Chen W. et al., 2023). Similarly, a flexible PLA membrane was developed through electrostatic spinning as a SERS-active substrate with screen-printed capture probes. The high surface area of spun filaments improves probe adherence compared to rigid substrates, enabling efficient target enrichment and sensitive detection. The membrane’s flexibility, durability, and biocompatibility suggest potential for large-scale production to meet high-throughput detection needs (Sun et al., 2024).

The merits of 2D SERS substrates ensure the consistent reproducibility and uniformity of the devised SERS platforms, wherein NPs serve as the foundational elements for electromagnetic field coupling, yielding stable composite structures with uniform distribution. This straightforward approach facilitates the creation of homogeneous structures exhibiting effective electromagnetic effects (Xiao et al., 2024; Yin et al., 2021). In summary, two-dimensional substrates exhibit superior performance, and the adoption of scaling strategies can mitigate background signal interference.

#### 2.2.3 Three-dimensional (3D) substrates

In contrast to 2D plasmonic substrates like nanomembranes and nanocavities, 3D substrates offer heightened SERS effects due to their substantial amplification of the incident electric field on three-dimensional nanostructures at the active SERS sites (Phan-Quang et al., 2019). Recent work devised a cost-effective and easily producible SERS platform comprising three-dimensional porous expanded graphite adorned with Ag NPs. The porous and highly three-dimensional architecture of the expanded graphite/Ag NPs nanocomposites facilitate rapid solvent infiltration, eradicate the coffee-ring effect, enable swift SERS measurements and owing to the extensive specific surface area, permit the decoration of additional metal NPs and adsorption of more analyte molecules (Çelİk and Kurt, 2021). Another study developed SERS substrates featuring silver-sealed poly (p-xylene C)-coated carbon NPs (Ag-PC@CNPs) through candle soot deposition, creating a porous carbon nanoparticle layer, followed by poly (p-xylene C) thin film deposition to shield the CNPs and ultimately silver nanoparticle sputtering. Operating akin to a nut guide, droplets rolling on the Ag-PC@CNP-coated substrate absorb the Ag-PC@CNPs, thereby engendering a self-collecting, highly sensitive SERS-activated droplet sensor with 3D hotspots (Li et al., 2020). Further research employed a deep eutectic solvent (DES) to fabricate plasmonic aerogels serving as sustainable SERS substrates, comprising heterogeneous structures of diverse gold NPs (AuNPs) synthesized in the presence of cellulose nanocrystals This analytical method relies on a 3D arrangement of AuNPs within the cellulose nanocrystal matrix, wherein interactions among transient cellulose nanocrystal collapse upon loading an aqueous analyte solution, forming requisite hotspots (Panikar et al., 2022).

Later, a novel 3D porous gold nanorod (Au NR) embedded in polyvinyl alcohol hydrogel is developed, featuring a multilayered backbone structure conducive to generating numerous SERS hot spots and regulating inter-particle nanogaps by adjusting gold nanorod density, effectively amplifying Raman signal (Shuyan et al., 2024). Using UV light to encapsulate cationic cellulose (CCNF) with Ag NCs in a polyacrylamide hydrogel, this CCNF serves to stabilize and align positively charged Ag NCs through its inherent positive surface charge and stability, ensuring a stable and uniform SERS signal. Notably, Ag NCs/CCNF/PAAM (polyacrylamide) hydrogels have excellent swelling and shrinking properties, which can effectively adsorb the test materials in the dissolved state and shrink in the dry state to reduce the nanoparticle interstitials and enrich the test materials, thus enhancing the SERS signals (Liu et al., 2023). The porous nature of the hydrogel exhibits selectivity towards substances in the plasma, streamlining the detection process by allowing for direct analysis of complex samples without intricate pretreatment.

In summary, 3D SERS substrates offer a greater specific surface area and a higher density of hotspots per unit area, thereby significantly enhancing the accessibility of analytes to the SERS active region. While the benefits of 3D SERS substrates are undeniable, the fabrication of top-tier 3D substrates is intricate, often demanding sophisticated and costly equipment as well as a series of intricate procedures (Phan-Quang et al., 2019). Looking ahead, it is foreseeable that more 3D structures boasting extensive surface areas and abundant hotspots will emerge, indicating the future trajectory of 3D SERS substrates.

### 2.3 The key role of artificial intelligence in SERS spectral analysis

The analysis of label-free SERS spectra is challenging due to their inherent complexity, high dimensionality, and subtle spectral variations arising from diverse biomolecular components. This complexity makes advanced computational analysis essential, as visual inspection of raw spectra is often inadequate (Bi et al., 2023; Lussier et al., 2020). Consequently, AI has become a cornerstone for interpreting these intricate datasets and achieving robust diagnostic classification.

A standard analytical pipeline integrates multiple computational techniques. Unsupervised methods like PCA are widely used for dimensionality reduction and visualizing inherent data structures. Subsequently, supervised algorithms—including LDA, SVM, RF, and PLS-DA—build predictive models to differentiate sample classes (e.g., cancer patients from healthy controls) with high specificity and sensitivity, as evidenced in numerous studies (Cao et al., 2022; Liang et al., 2020; Peng et al., 2023; Vargas-Obieta et al., 2016).

More recently, deep learning architectures such as CNN and DNN have demonstrated the capability to automate feature extraction by learning directly from raw or minimally processed spectral data, facilitating end-to-end classification. The integration of these AI techniques has significantly enhanced the objectivity and accuracy of label-free SERS, solidifying its position as a powerful platform for clinical liquid biopsy (Cheng et al., 2021b; Nguyen et al., 2024; Shin et al., 2023).

A survey of the studies presented in this review (Table 1) indicates that the field has not converged on a single optimal machine learning algorithm for label-free SERS analysis. Instead, a diverse toolkit of algorithms is being successfully employed. The selection often reflects a trade-off between model interpretability and predictive power. Traditional methods like PCA-LDA are widely adopted, likely due to their effectiveness in capturing latent variables in spectral data and providing a transparent decision process, which is valuable for initial biomarker discovery (Li J. et al., 2023; Vargas-Obieta et al., 2016). In contrast, deep learning models (e.g., CNN, DNN), while often acting as ‘black boxes’, are increasingly applied to tackle more complex pattern recognition tasks, such as multi-cancer classification or predicting the tissue-of-origin from exosomal SERS profiles, where their ability to autonomously extract hierarchical features offers a potential advantage (Lin X. et al., 2021; Shin et al., 2023). Future work featuring direct, head-to-head comparisons of multiple algorithms on standardized SERS datasets will be invaluable to definitively guide algorithm selection. Concurrently, advancing the quantitative robustness of SERS through innovative substrate and assay engineering, such as spatial-temporal encoding strategies for high-throughput biosensing (Long et al., 2025), represents a complementary and critical direction for the field.

**TABLE 1:** Summary of algorithm analysis techniques used by SERS strategies.

Analyte	Cancer	SERS platform	Method(s) used	References
Mouse serum	Lung cancer	Au NSs	PCA-RCKNCN	Cai et al. (2022)
Human plasma exosomes	Lung cancer	2D substrates Au NPs	Deep learning	Cao et al. (2022)
Human serum	Breast cancer	Au NPs	PCA-LDA	Vargas-Obieta et al. (2016)
Human plasma	Breast cancer	3D-PC Au NPs	CNN	Nguyen et al. (2024)
Human serum exosomes	Breast cancer	2D substrates Au NSs	ANN	Xie et al. (2022)
Human serum	Liver cancer	3D ZnO-Ag NPs	DNN	Cheng et al. (2021a)
Human serum	Liver cancer	3D ZnO-Ag NPs	CNN	Cheng et al. (2021b)
Human serum	Liver cancer	Ag NPs	PLS-LDA	Li et al. (2022)
Human serum	Colon cancer	Ag NPs	DT, RF, and PCA–LDA	Peng et al. (2023)
Human serum	Colon cancer	2D substrates Ag NPs	PCA-LDA	Li et al. (2023c)
Human serum	Colon cancer	3D substrates Ag NPs	PCA-LDA	You et al. (2023)
Human plasma	Prostate cancer	Ag NPs	PCA-MLP	Ge et al. (2024)
Human serum	Prostate cancer	Ag NPs	CNN	Shao et al. (2020)
Mouse serum	Gastric cancer	2D substrates Au NHs	PCA -TLNN	Cao et al. (2023)
Human plasma	Thyroid cancer	Ag NPs	Lasso-PLS-DA	Liang et al. (2020)
Human serum	Thyroid cancer	Ag NPs	PLS, LDA	Xia et al. (2021)
Human serum	Cervix	Au NPs/785 PSi PhCs	PCA-LDA, PCA-SVM	Gao et al. (2023)
Human serum	Leukaemia, hepatitis B virus and breast cancer	2D substrates Ag NPs	Deep learning	Lin et al. (2021a)
Human serum	Liver cancer, Prostate cancer	Ag NPs	PLS-SVM	Bai et al. (2022)
Human serum exosomes	Breast cancer, Cervix	3D substrates Au NPs	PCA-LDA	Diao et al. (2023)
Human plasma exosomes	Lung, breast, colon, liver, pancreas and Gastric cancer	2D substrates Au NPs	Deep learning	Shin et al. (2023)

PCA-RCKNCN, principal component analysis - revised constrained k-nearest neighbor classification; PCA-LDA, principal component analysis-linear discriminant analysis; 3D-PC, 3D photonic crystal; CNN, convolutional neural network; ANN, artificial neural network; DNN, deep neural network; PLS-LDA, partial least squares-linear discriminant analysis; PCA -TLNN, principal component analysis-two layer nearest neighbor; Lasso-PLS-DA, lasso-partial least squares discriminant analysis; PCA-SVM, principal component analysis - support vector machine.

The widespread application and performance of these techniques across various cancers are comprehensively summarized in Table 1.

## 3 SERS marker-free detection in liquid biopsy

### 3.1 SERS marker-free detection in blood

Blood contains a complex mixture of cells, biomolecules, and biochemical markers that can provide valuable diagnostic information for cancer detection. This section specifically examines label-free SERS approaches for analyzing blood-based liquid biopsies, which offer non-invasive alternatives to traditional tissue biopsies. Three primary sample types are discussed: plasma, serum, and isolated exosomes, with their respective applications and analytical considerations summarized in Table 1.

#### 3.1.1 Application in lung cancer

**FIGURE 2:**
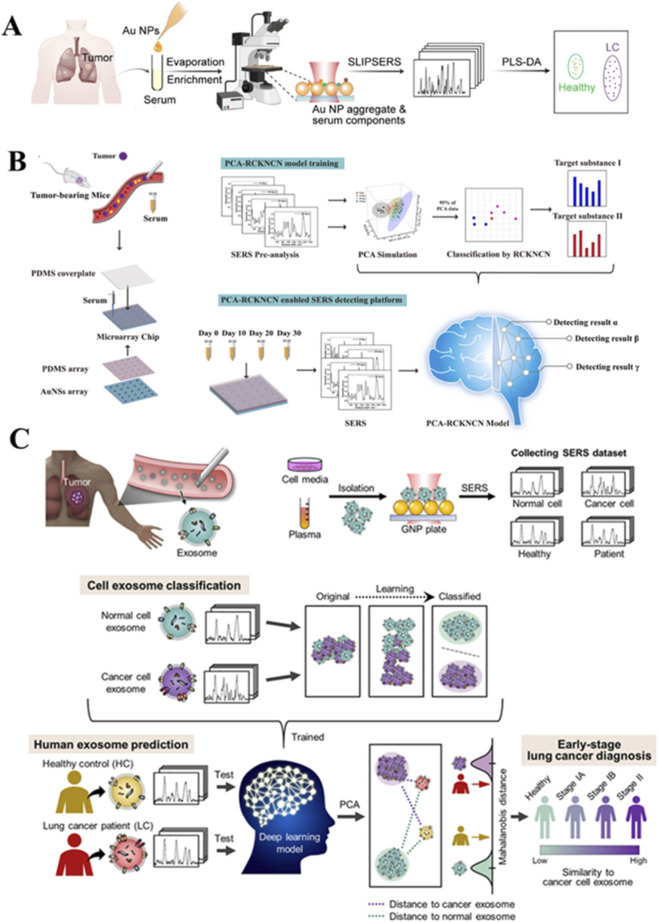
Schematic illustrations of SERS-based label-free detection methods for lung cancer diagnosis using blood components. **(A)** Label-free serum detection utilizing gold NPs for the diagnosis of lung cancer. Reproduced with permission from ref (Cai et al., 2022). **(B)** SERS combined with PCA-RCKNCN for rapid and precise identification of lung cancer using serum. Reproduced with permission from ref (Cao et al., 2022). **(C)** Early diagnosis of lung cancer through deep learning-enabled spectral analysis of circulating exosomes. Reproduced with permission from ref (Shin et al., 2020).

Lung cancer remains a leading cause of cancer-related deaths globally, highlighting the urgent need for early detection methods. The evolution of label-free SERS liquid biopsy for lung cancer reflects a strategic shift from analyzing complex, bulk biofluids toward targeting increasingly specific and rich biomarker subpopulations. This progression, from whole serum to exosomes and further to circulating tumor cells (CTCs), aims to enhance diagnostic specificity and gain deeper biological insights. Blood-based SERS analysis has gained traction for lung cancer screening based on these biomarker classes (Figure 2). For instance, one label-free SERS strategy uses minimal serum volumes (1 μL) to rapidly capture diagnostic spectral fingerprints. Combined with chemometric analysis, this approach successfully distinguishes lung cancer patients (n = 33) from healthy controls (n = 23) with high accuracy (Cai et al., 2022).

By combining gold nanostar-coated microarray chips with a machine learning algorithm (PCA-RCKNCN) to analyze serum from tumor-bearing mice, researchers achieved 100% accuracy in staging lung tumors (Cao et al., 2022).

While serum analysis provides a broad diagnostic overview, the pursuit of higher specificity has driven research toward exosomes—nanoscale vesicles that carry molecular cargo (e.g., proteins, nucleic acids) specifically reflective of their parent tumor cells. The development of SERS-based biosensors for exosome detection has seen significant progress (Li et al., 2021). Exosomes are cellular extracellular vesicles found in blood with sizes at nanometer scales (50∼90 nm). With exosomes as carriers of intercellular communication, it can be used as non-invasive surrogate marker of cancer. Via comprehensive analysis of the entire SERS profile of exosomes, those exosomes derived from lung cancer cells were distinguished from those of normal cells, with an impressive sensitivity of 95.3% and specificity of 97.3% (Park et al., 2017). Through deep learning-based SERS analysis of exosomes, independent investigations show the accurate diagnosis of early-stage lung cancer (Figure 2C). The deep learning model, trained on SERS signals from exosomes of both normal and lung cancer cell lines, achieves a classification accuracy of 95.0%. In a cohort of 43 patients (including those with stage I and II cancers), the model identifies that 90.7% of patients’ plasma exosomes show stronger spectral alignment with lung cancer cell exosomes than with healthy controls. The model demonstrates an area under the curve (AUC) of 0.912 for lung cancer diagnosis and an AUC of 0.910 for the entire cohort, including stage I patients (Shin et al., 2020).

To analyze plasma and exosomes from healthy controls, adenocarcinoma *in situ*, and invasive adenocarcinoma, authors introduced a SERS “Combined Spectrum,” integrating spectral data from both biomarkers (Zhang et al., 2024). Four machine learning algorithms achieved >97.0% accuracy and AUC >0.95, demonstrating high diagnostic potential. This non-destructive approach, utilizing gold NPs without specific markers, shows promise for early lung cancer screening and could potentially be applied to other diseases. Recently, the same team developed a refined SERS approach for early lung cancer detection, addressing key limitations of conventional methods such as inconsistency and prolonged processing times. Because critical biomarkers are often masked by dominant uric acid signals, the team introduced a three-step pretreatment protocol: plasma dilution, weak acidification (pH adjustment), and stabilization with liquid paraffin. In combination with Ag NPs, this protocol enhances spectral clarity, suppresses uric acid interference, and amplifies disease-specific signals. Via multivariate PCA-LDA analysis, authors demonstrate a diagnostic accuracy of 91.7%, with further optimization yielding an AUC of 0.975. This streamlined, liquid-phase SERS strategy significantly improves reproducibility and clinical feasibility, offering a practical platform for minimally invasive lung cancer screening. The authors suggest future cross-validation with complementary techniques to refine spectral interpretation and expand clinical applications (Lu et al., 2025).

Building on the concept of analyzing tumor-derived materials, the most direct approach is the physical detection and characterization of circulating tumor cells (CTCs) themselves. To detect circulating tumor cells (CTCs), researchers have integrated dual-modal SERS bioprobes with advanced machine learning techniques. This cutting-edge methodology leverages the exceptional sensitivity of SERS bioprobes, achieving a remarkable detection limit of 2 cells/mL for CTC identification. Furthermore, the implementation of sophisticated machine learning models, including PCA and Random Forest algorithms, has yielded an outstanding 98.0% accuracy in discerning CTCs from white blood cells. Notably, this strategy also exhibits an exceptionally high recognition sensitivity specifically for A549 lung cancer cells. By harmonizing efficient magnetic separation with unparalleled specificity, this approach significantly mitigates subjective interferences, underscoring the immense potential of encoded SERS bioprobes for CTC detection. Such advancements not only promise significant improvements in cancer diagnosis but also pave the way for future applications in CTC subtyping (Zhang et al., 2025).

Collectively, reports demonstrate SERS-based liquid biopsy for the early detection of lung cancer. The combination of label-free SERS analysis of serum, exosomes, and CTCs with machine learning achieves diagnostic accuracies exceeding 90.0% in controlled studies. Particularly noteworthy is the consistent performance across different biomarker classes (AUC 0.9–1.0), though clinical translation requires addressing several challenges: (1) standardization of sample preparation protocols, (2) validation in larger, diverse cohorts, and (3) integration with existing diagnostic workflows. Future research directions should prioritize technical refinements to improve reproducibility while maintaining the technique’s inherent advantages of rapidity and minimal sample requirements.

#### 3.1.2 Application in breast cancer

According to statistics of World Health Organization in 2022, breast cancer was the most common cancer of women in 157 out of 185 countries. Early diagnosis of breast cancer significantly improves clinical outcomes. Liquid biopsy techniques analyzing tumor-derived biomarkers in bodily fluids show particular promise for early detection. Blood-based SERS analysis has emerged as a powerful platform for label-free breast cancer diagnosis.

**FIGURE 3:**
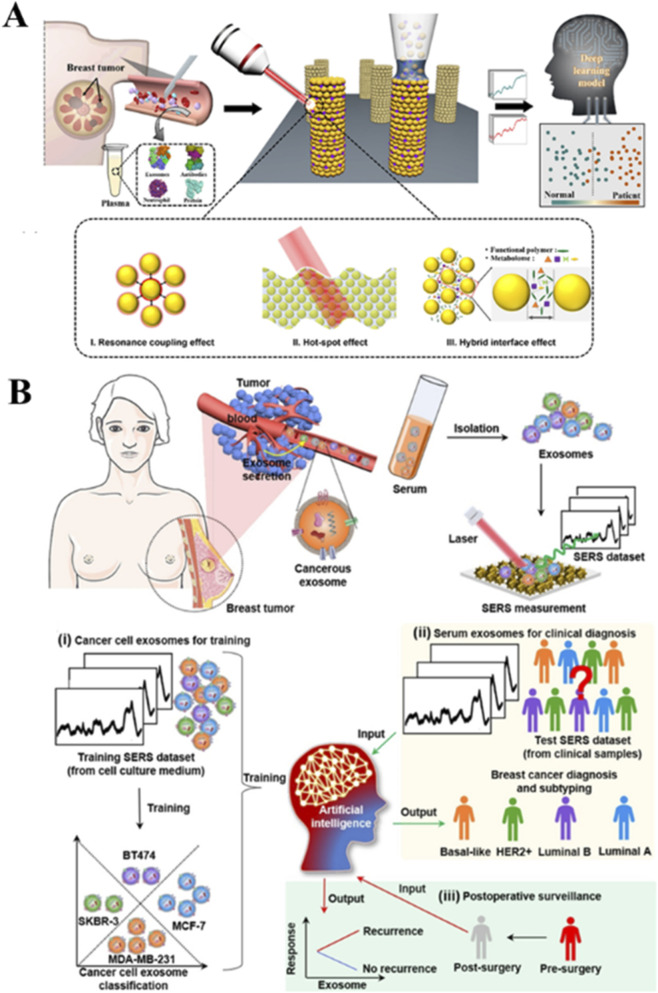
SERS-based liquid biopsy strategies for breast cancer detection using blood-derived biomarkers. **(A)** 3D plasmonic supercluster platform for serum analysis. Data adapted from reference (Nguyen et al., 2024). **(B)** AI-assisted exosome SERS analysis for diagnosis and treatment monitoring. Data adapted from reference (Xie et al., 2022).

When combined with PCA-LDA, serum SERS analyses achieve 96.0% sensitivity and 87.0% specificity in distinguishing breast cancer patients from healthy controls (Vargas-Obieta et al., 2016). Subsequent work on clinical-stage classification using serum SERS with partial least squares discriminant analysis (PLS-DA) analysis show 90% sensitivity and 98.4% specificity, with an AUC value of 0.94 (Nargis et al., 2020). Another method employing serum protein SERS analysis with PLS-SVM modeling demonstrates 90.00% sensitivity and 97.78% specificity, achieving an ROC of 0.940 (Lin Y. et al., 2021). In combination of 3D-PC SERS substrates combined with deep learning, authors achieve 93.4% classification accuracy between healthy and cancerous samples (Nguyen et al., 2024) (Figure 3A). When focusing on serum exosomes, AI-driven SERS analyses demonstrate 100% prediction accuracy for untreated breast cancer patients across various subtypes. This approach enables surgical outcome evaluation through PCA-based similarity analysis (Xie et al., 2022) (Figure 3B).

The exosome-based SERS analysis has recently found success for predicting neoadjuvant therapy outcomes in HER2-positive breast cancer. A machine learning model combining PCA, LDA, and SVM achieved >0.89 AUC in analyzing the molecular changes of serum exosomes, with accuracy improving to >94.0% when incorporating a HER2-specific exosome capture system (Jia et al., 2025). Parallel developments in subtype detection have yielded a dual-modal SERS-fluorescence approach using hexoctahedral Au NPs, demonstrating 94.0% classification accuracy for breast cancer subtypes (AUC ≥0.98) through symphonic SERS spectra analysis and LDA modeling (Hu et al., 2025).

#### 3.1.3 Application in liver cancer

**FIGURE 4:**
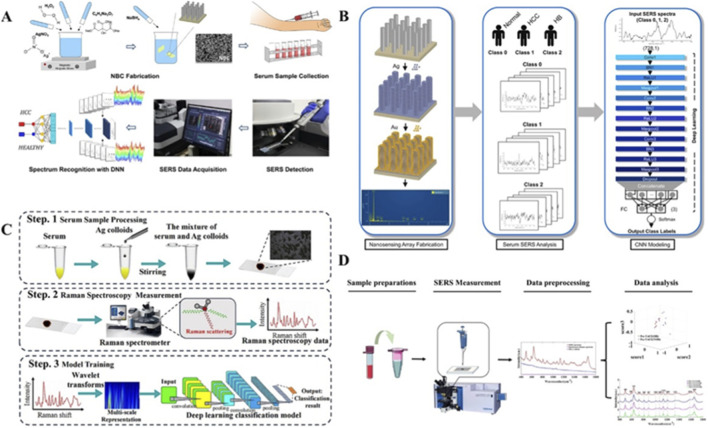
SERS-based liquid biopsy approaches for liver cancer diagnosis and monitoring. **(A)** Schematic of nanoplasmonic biosensing chip for antibody-free HCC screening using spectral deep learning (Cheng et al., 2021a). **(B)** Integrated biosensing platform combining SERS detection with CNN classification for liver disease serology (Cheng et al., 2021b). **(C)** Multi-scale deep learning framework for HCC detection through SERS spectral analysis (Yang et al., 2024). **(D)** SERS-based early assessment of chemotherapy response in HCC patients (Li et al., 2022).

In Asia, liver cancer is the second leading cause of malignant death and the fifth most common cancer type, with 72.5% of the world’s cases in 2020 (Fu et al., 2025). To detect hepatocellular carcinoma (HCC), a nano-plasmonic biosensing chip platform was developed and later found broad applicability across cancer types. This approach requires only one blood drop for SERS detection (Figure 4A). When combined with deep neural networks, it achieved 91.0% accuracy in distinguishing HCC from healthy controls, validated through independent testing (Cheng et al., 2021a). This foundational work established the technical feasibility of minimal-sample liquid biopsy for hepatic malignancies. Building upon this work, the research team engineered an advanced nano-sensing array serving as a high-throughput SERS sensor (Figure 4B). Their methodology involved direct analysis of serum samples from three clinical groups: healthy controls, hepatitis patients, and HCC cases. By employing a CNN classifier, the system attained 97.78% classification accuracy on an independent validation dataset, showcasing its potential for clinical implementation (Cheng et al., 2021b).

In a complementary approach, researchers implemented a sophisticated analytical pipeline combining wavelet transform-based multi-scale feature extraction with neural network classification (Figure 4C). Their model, trained on SERS spectra from liver cancer patients and healthy volunteers, achieved outstanding diagnostic performance with 99.38% accuracy, 99.8% sensitivity, and 97.0% specificity. This represents one of the most accurate SERS-based liver cancer detection systems reported to date (Yang et al., 2024).

For therapeutic monitoring applications, investigators explored the prognostic potential of serum SERS in HCC patients undergoing chemotherapy (Figure 4D). Their longitudinal study identified significant spectral changes in circulating nucleic acids and amino acids within just 3 days post-treatment. By developing a PLS-LDA prognostic model, they achieved prediction accuracy ranging from 88.24% to 100%, demonstrating the technology’s capability for real-time treatment response assessment (Li et al., 2022).

#### 3.1.4 Application in colon cancer

**FIGURE 5:**
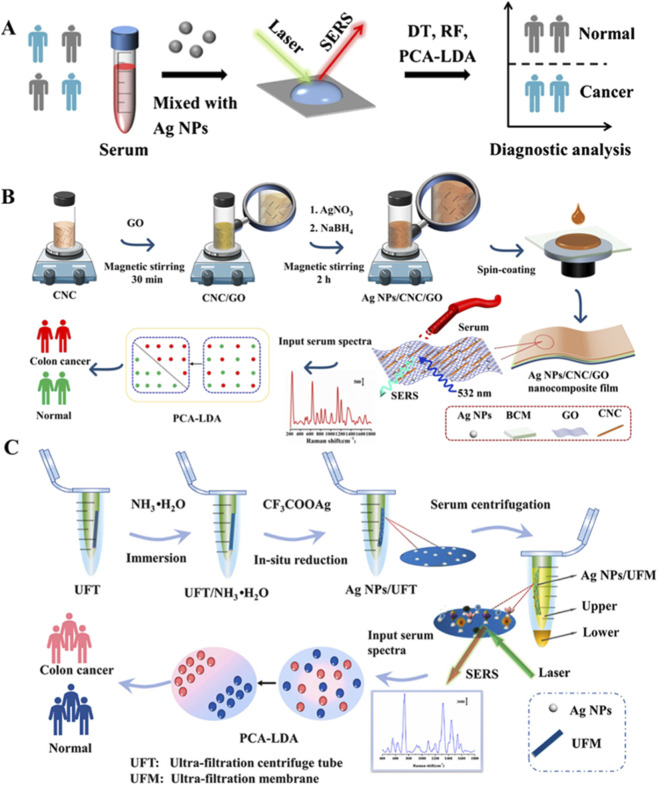
Technical schematics of SERS-based detection platforms for colorectal cancer diagnosis. **(A)** Machine learning framework for spectral analysis of serum samples (Peng et al., 2023). **(B)** Nanocomposite membrane architecture for SERS substrate fabrication (Li J. et al., 2023). **(C)** Microfluidic biosensor design integrating *in situ* nanoparticle synthesis NPs (You et al., 2023).

Colorectal cancer is the second leading cause of malignant deaths and the third most commonly diagnosed cancer globally. In 2022, about 1.93 million patients were diagnosed with colorectal cancer worldwide (Zhang et al., 2022). Recent advances in SERS-based liquid biopsy have demonstrated significant progress in colorectal cancer detection through multiple methodological approaches. A label-free serum SERS assay incorporating internal standard calibration and machine learning analysis achieved diagnostic accuracy exceeding 90.0% with 100% specificity in clinical validation studies involving balanced cohorts of cancer patients and healthy controls (Peng et al., 2023) (Figure 5A). Alternative methodologies utilizing homogeneous SERS-active nanomembranes for serum analysis have shown promising results, with reported classification accuracy of 84.1% accompanied by 89.3% specificity and 71.4% sensitivity when combined with PCA-LDA modeling (Li J. et al., 2023) (Figure 5B).

Technical innovations in biosensor design have successfully addressed several challenges in liquid-phase SERS detection, particularly regarding nanoparticle stability and signal specificity. Integrated biosensor systems capable of stabilizing colloidal substrates while selectively concentrating diagnostic biomarkers have demonstrated 87.5% accuracy with complete specificity in clinical validations (You et al., 2023) (Figure 5C).

The consistent application of machine learning algorithms, particularly PCA-LDA, has proven effective for spectral pattern recognition across these diverse methodologies. Current research efforts are focused on protocol standardization, clinical cohort expansion, and development of multiplexed detection platforms to address remaining challenges in reproducibility and comprehensive biomarker analysis. The demonstrated technical capabilities, particularly the repeated achievement of complete specificity across multiple studies, indicate strong potential for clinical translation in non-invasive cancer screening applications.

Recent advances in exosome analysis have demonstrated the effectiveness of integrating Raman spectroscopy with machine learning algorithms for cancer detection. This combined analytical approach, utilizing a PCA-LDA framework, achieved 93.3% classification accuracy for exosomes derived from multiple cancer cell lines (Villazon et al., 2025). The methodology showed particularly robust performance in identifying COLO205 colorectal cancer exosomes, with an F1-score of 98.2%, while also revealing distinct lipid profiles that could serve as potential cancer-specific biomarkers. These findings highlight the synergistic potential of spectroscopic techniques coupled with computational analysis for developing sensitive liquid biopsy platforms.

#### 3.1.5 Application in prostate cancer

**FIGURE 6:**
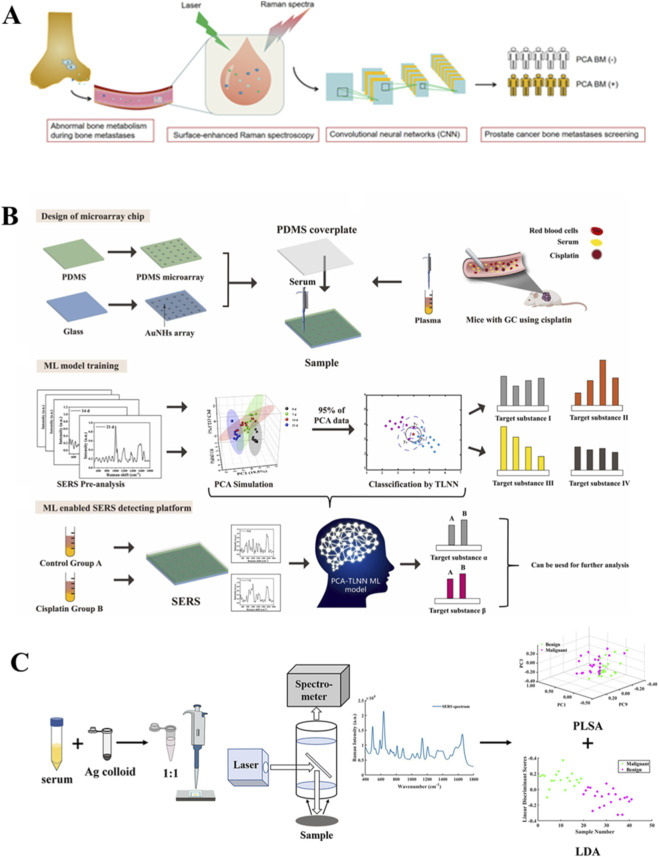
SERS applications in cancer diagnostics through blood component analysis. **(A)** CNN-based detection of prostate cancer bone metastases from serum spectra (Shao et al., 2020). **(B)** Treatment response monitoring in gastric cancer via PCA-TLNN spectral analysis (Cao et al., 2023). **(C)** Thyroid nodule classification using serum SERS profiling (Xia et al., 2021).

Prostate cancer is the fourth most common cancer globally and the second most common cancer in men. A combined PCA-MLP approach analyzing plasma samples of prostate cancer patients achieved 96.70% classification accuracy, establishing baseline performance for primary cancer detection (Ge et al., 2024). Subsequent advancements utilizing convolutional neural networks show expanded clinical applications - a LeNet-5 CNN architecture analyzing serum spectra attained 81.70% accuracy (80.63% sensitivity, 82.82% specificity) for bone metastasis identification (Shao et al., 2020) (Figure 6A), while alternative CNN implementations reached 85.14% accuracy in differentiating prostate cancer from benign prostatic hyperplasia with additional Gleason score prediction capability (Wang Y. et al., 2023). These approaches consistently maintain >80.0% accuracy across diverse sample types and clinical applications, though performance variations reflect methodological differences in spectral processing and neural network architectures. The demonstrated technical capabilities suggest particular promise for complex diagnostic challenges like metastatic screening and tumor grading, pending resolution of standardization needs in spectral acquisition and validation in broader patient cohorts. Future integration with existing diagnostic markers may further enhance clinical utility while addressing current specificity limitations.

#### 3.1.6 Application to gastric cancer, thyroid and cervical cancer

For monitoring cisplatin-treated gastric cancer, a SERS approach combining PCA -TLNN was developed using microarray chips with Au nano hexagonal (AuNH) substrates to analyze sera from treated gastric cancer mice at various treatment stages (Figure 6B). This method achieved prediction accuracy, sensitivity and specificity of 97.5%, 90.0% and 96.7%, respectively (Cao et al., 2023).

In thyroid tumor diagnostics, a membrane filtration SERS technique was introduced to analyze plasma ultrafiltrates from 102 patients (70 carcinomas, 32 benign tumors). After background correction and normalization, SERS spectra were processed using Lasso-PLS-DA incorporating first-order derivatives. The model yielded 90.2% diagnostic accuracy, with 97.1% sensitivity and 75.0%, specificity (Liang et al., 2020). In a separate investigation, a research group used serum SERS to analyze samples from 22 healthy volunteers, 19 benign nodules and 22 malignant nodules (Figure 6C). Using combination of PLS-LDA, they achieved 93.65% accuracy (healthy vs. nodules) and 82.93% accuracy (benign vs. malignant), with corresponding sensitivity of 92.68%/81.82% and specificity of 95.45%/84.21% (Xia et al., 2021).

For cervical cancer detection, an optimized SERS protocol using gold NPs/785 porous silicon photonic crystals (Au NPs/785 PSi PhCs) was developed for low-concentration. Combining PCA, LDA and SVM, the optimized model achieved 97.9% and 96.9% accuracy for different classification tasks (Gao et al., 2023).

#### 3.1.7 Application to identification of multiple cancers

**FIGURE 7:**
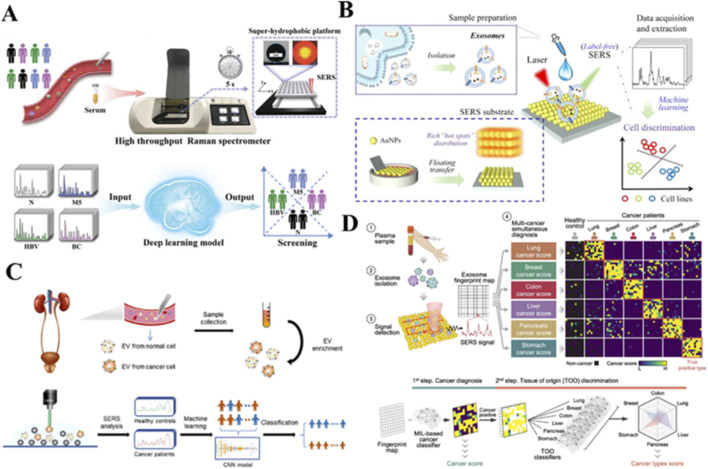
SERS-based liquid biopsy approaches for cancer diagnosis. **(A)** DCNN analysis of serum Raman spectra for prostate cancer metastasis detection (Lin X. et al., 2021). **(B)** 3D plasmonic nanostructures for urinary exosomal miRNA profiling in prostate cancer (Diao et al., 2023). **(C)** PCA-TLNN platform for monitoring cisplatin-treated gastric cancer (Qian et al., 2022). **(D)** Serum SERS analysis for thyroid nodule classification (Shin et al., 2023).

Recent studies demonstrate SERS technology’s capability for multiplex cancer detection. One approach using gold NPs/785 porous silicon photonic crystals (Au NPs/785 PSi PhCs) achieved 97.77% accuracy in differentiating healthy individuals, cervical cancer and breast cancer patients through multivariate statistical analysis (Gao et al., 2021). To tackle a more complicated distinguishment from a diverse cohort consisting of 203 healthy volunteers, 77 leukemia M5 patients, 94 hepatitis B virus patients and 321 breast cancer patients, researchers introduced a fully automated superhydrophobic platform tailored for SERS applications. They acquired a substantial dataset comprising 695 high-quality serum SERS spectra (Figure 7A). Leveraging serum SERS signals from both normal and patient groups, their deep learning model achieved maximum classification accuracy of 100%. Furthermore, when combined with SERS, this deep learning model exhibited outstanding diagnostic accuracy of 98.6% against an independently maintained test set (Lin X. et al., 2021).

To distinguish plasma samples from 26 bladder cancer patients, 38 kidney cancer patients and 39 normal subjects, authors use three distinct algorithms—PCA-LDA, partial least squares random forest (PLS-RF) and PLS-SVM to analyze high-quality SERS signals. They achieved classification accuracies of 98.1%, 100% and 100%, respectively, for differentiating SERS spectra of plasma from cancer patients and normal subjects. Additionally, the classification accuracies of these algorithms for distinguishing between bladder and kidney cancers stood at 81.3%, 91.7% and 98.4%, respectively (Bai et al., 2022).

Recent methodological advances in SERS-based cancer detection show two distinct technical pathways. For serum analysis, a label-free SERS serological strategy targeting sera from liver cancer patients (n = 40), prostate cancer(n = 32) and healthy volunteers (n = 30). Employing PLS and SVM algorithms, they constructed a diagnostic model capable of classifying SERS spectral data. The accuracy of differential diagnosis between the normal group and the two types of cancers simultaneously reached an impressive 98.04%. Furthermore, for the unknown test set, PLS-SVM achieved 100% accuracy in distinguishing between cancer and normal groups (Gao et al., 2022).

For exosome analysis, a machine learning-based SERS approach using 3D plasmonic AuNP nanomembranes demonstrated 91.1% accuracy for cell line-derived exosomes and 93.3% for clinical samples (Diao et al., 2023) (Figure 7B). Another study applying CNN analysis to serum exosomes reported diagnostic accuracies of 79.3% (prostate), 78.7% (renal) and 74.2% (bladder) with AUCs of 0.80–0.88 (Qian et al., 2022) (Figure 7C). The most advanced platform achieved pan-cancer detection (AUC = 0.970) with 90.2% sensitivity and 94.4% specificity, though tumor origin prediction accuracy was 72% (Shin et al., 2023) (Figure 7D).

Future directions should focus on: 1) Standardization - establishing cross-platform substrate fabrication protocols (e.g., AuNH, porous photonic crystals) and unified data acquisition procedures; 2) Clinical translation - conducting multicenter validation for prevalent cancers in China (e.g., gastric/liver cancers) and developing portable devices; 3) Mechanistic studies - deciphering correlations between exosomal SERS signatures and tumor heterogeneity, particularly addressing the current limitation in organ-of-origin prediction accuracy (e.g., 72.0%) through integration with proteomics and other omics data. These advancements will position SERS as a clinically viable universal liquid biopsy platform.

### 3.2 Urine detection

Because urine is from blood, presumably its chemical composition is comparable to that of plasma. Therefore, the label-free urine SERS strategy is similar to that for blood. This section will focus on SERS label-free liquid biopsy strategies for analyses of urine and its exosomes, summarized in Table 2.

**TABLE 2:** Summary of algorithm analysis techniques adopted by SERS strategies.

Analyte	Cancer	SERS platform	Method(s) used	References
Human urine	Prostate cancer	Au NPs	PCA-LDA	Del Mistro et al. (2015)
Human urine	Pancreatic and prostate cancer	3D substrates Ag NWs	OPLS-DA	Phyo et al. (2021)
Human urine	Breast cancer	Ag NPs	PCA-LDA	Moisoiu et al. (2019)
Mouse urine	Bladder cancer	3D ZnO-Au NPs	PLS-DA	Lee et al. (2024)
Human urine	Prostate and pancreatic cancer	3D substrates Au NPs	RNN,CNN	Linh et al. (2023)
Human salivary exosomes	Oral cancer	Ag NPs	PCA-LDA	Faur et al. (2023)
Human saliva	Oral cancer	Ag NPs	PCA-LDA	Borşa et al. (2023)
Human saliva	Lung cancer	AuNPs	PCA-SVM	Karunakaran et al. (2022)
Human saliva	Lung cancer	3D-PHP AuNPs	LR	Linh et al. (2024)
Human salivary exosomes	Gastric cancer	Au NCs	LDA	Liu et al. (2022)
Human sweat	Gout	2D substrates Ag NWs	ANN	Chen et al. (2024)
Human tears	Breast cancer	2D substrates Au/HCP-PS	PCA-LDA	Kim et al. (2020)

**FIGURE 8:**
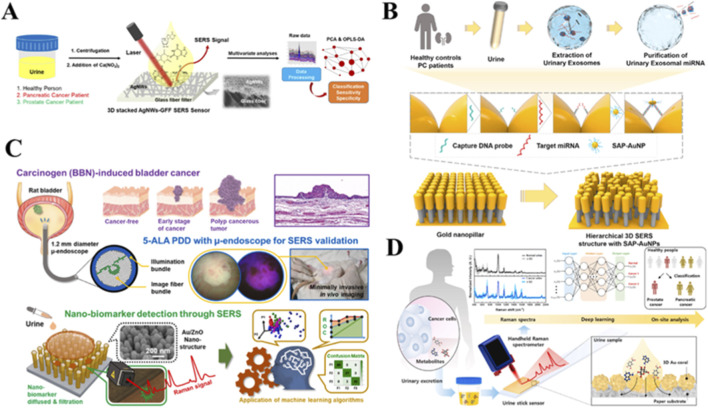
SERS-based liquid biopsy approaches for cancer diagnosis. **(A)** DCNN analysis of serum Raman features for prostate cancer metastasis detection (Phyo et al., 2021). **(B)** 3D plasmonic nanostructures for urinary exosomal miRNA detection in prostate cancer (Kim et al., 2022). **(C)** SERS-ML integration for bladder cancer diagnosis in rodent models (Lee et al., 2024). **(D)** 3D nanostructured paper for urine sensing with DL-assisted cancer screening (Linh et al., 2023).

SERS spectra of urine samples from prostate cancer patients and healthy donors were analyzed by PCA-LDA. Authors obtain a classification model with 100% sensitivity, 89.0% specificity and 95.0% overall diagnostic accuracy (Del Mistro et al., 2015). To assess the response to treatment of prostate cancers in a cohort of recurrent and non-recurrent patients, SERS spectra of the patients’ urine were obtained with genetic algorithm-partial least squares-linear discriminant analysis (GA-PLS-LDA). The accuracy in distinguishing between recurrent and non-recurrent cohorts was 86.6%, sensitivity 86.0% and specificity 87.1% (Ma et al., 2021). SERS spectra of urine collected by 3D stacking of Ag NWs on a GFF sensor was used for diagnosing of pancreatic and prostate cancer. Authors then used the orthogonal partial least squares discriminant analysis (OPLS-DA) method to analyze these SERS spectral patterns. OPLS-DA had 100% sensitivity and 100% specificity in the pancreatic and prostate cancer groups and 100% sensitivity and 100% specificity in discriminating between the normal control and combined cancer groups (Phyo et al., 2021) (Figure 8A).

Using SERS, the research team conducted an analysis of urine samples from 53 breast cancer patients and 22 healthy volunteers, applying multivariate data analysis techniques to discern between the two cohorts. Through their PCA-LDA approach, the SERS spectra of urine samples from breast cancer patients were categorized with 81.0% sensitivity, 95.0% specificity and an overall accuracy of 88.0% (Moisoiu et al., 2019). In a separate study, the team developed a quantitative, label-free miRNA sensing platform for detecting urinary exosomal miRNAs using SERS. This innovative platform relies on a sophisticated 3D layered plasmonic nanoarchitecture, formed through self-assembly processes involving target complementary DNA probe-conjugated gold NPs and head-embedded gold nanopillars in the presence of target miRNAs (Figure 8B). The resulting nanostructure generated numerous 3D plasmonic hotspots, enabling significant SERS signal amplification. By analyzing the SERS spectra of clinical urine samples, the researchers achieved a remarkable diagnostic accuracy of 0.93 in distinguishing prostate cancer patients from healthy controls (Kim et al., 2022).

Beyond detecting bladder cancer using urine supernatant and sediment, efforts for prediction of tumor grade were also achieved. Combining the results of the two urine components, the overall diagnostic sensitivity and specificity of SERS for high-grade tumors were 100% and 98.85% and for low-grade tumors were 97.53% and 90.80% (Hu et al., 2021). To achieve early, precise, label-free and non-invasive diagnosis of bladder tumors, SERS analyses reveal nano biomarkers in urine droplets. In a rat model induced with n-butyl-N-4-hydroxybutylnitrosamine, tumor progression (including cancer staging and polypoid tumor formation) was monitored in a minimally invasive fashion using a compact 1.2 mm endoscope. Using a gold-plated zinc oxide nanoporous chip, a drop of urine from each group of cancer-free, early-stage and polyp-type cancer underwent nano biomaterial filtration to selectively enhance Raman signals of nanoscale analytes. Subsequently, acquired Raman spectra were analyzed using PCA and partial least squares discriminant analysis to identify diagnostic clusters based on labeled samples (Figure 8C). Through the integration of SERS and machine learning, a diagnostic accuracy of ≥99.6% was achieved for diagnosing bladder tumors in the early and polyp stages (Lee et al., 2024).

Designed for the diagnosis of prostate and pancreatic cancer using human urine, a novel three-dimensional plasmonic coral nanostructure was developed as a SERS substrate (Figure 8D). Then, two distinct deep learning models: recurrent neural network (RNN) and CNN—were then used to analyze SERS spectra. The RNN binary classification model demonstrated an AUC of 0.9997, 99.4% sensitivity, 100% specificity and 99.7% accuracy according to the confusion matrix. On the other hand, the CNN multi-class classification model achieved a remarkable 96.8% sensitivity in the test dataset, with specificities of 99.6% (prostate) and 97.7% (pancreas) and an overall accuracy of 96.8% (Linh et al., 2023). This innovative approach suggests the potential of coupling urine test strips with handheld Raman spectroscopy as a promising diagnostic platform for analyzing various human body fluids in the future.

### 3.3 Saliva detection

**FIGURE 9:**
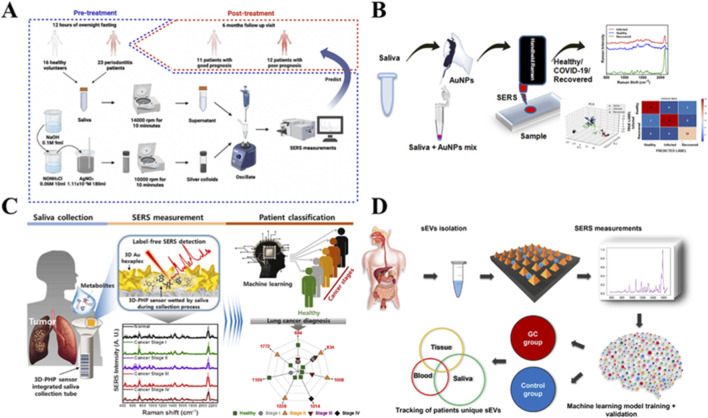
SERS-based saliva analysis for disease diagnosis. **(A)** Periodontal treatment prognosis using pretreatment salivary SERS (Chen S. et al., 2023). **(B)** COVID-19 detection through AI-classified salivary SERS fingerprints (Karunakaran et al., 2022). **(C)** On-site breast cancer biosensors via salivary SERS (Linh et al., 2024). **(D)** Gastric cancer detection using gold nanopyramid arrays for extracellular vesicle analysis (Liu et al., 2022).

Human saliva presents its unique wealth of biomolecules, proteins and metabolites that act as surrogate markers for disease and health. Saliva offers an ideal medium for disease diagnosis due to its simplicity, non-invasiveness, and convenient collection. It is particularly helpful for large-scale health surveys. To distinguish non-surgical periodontal therapy in patients with periodontitis from healthy controls, recent research used “leave-one-out cross-validation” (iteratively testing single samples against the remaining training set) (Figure 9A). This research demonstrates diagnostic accuracy, sensitivity and specificity of 87.18%, 86.96% and 87.50%, respectively. The method showed consistent performance for discriminating between good and poor prognosis groups (86.96% accuracy, 83.34% sensitivity and 90.90% specificity). The AUC values were 0.9592 for distinguishing the periodontitis group from the control group and 0.8485 for prognosis prediction (Chen S. et al., 2023).

Another study employed SERS to capture vibrational spectra of salivary exosomes isolated from patients with oral cavity and oropharyngeal squamous cell carcinoma, as well as healthy controls. Discrimination between malignant and non-malignant samples was performed using PCA-LDA, showing sensitivity of up to 79.3% between the two groups, though varying by spectral interval used for multivariate analysis (75.9% sensitivity for full range spectra) (Faur et al., 2023). Further work on filtered saliva samples yielded an accuracy of 77.0%, with sensitivity and specificity of 71.0% and 83.0%, respectively, for oral cancer diagnosis (Borşa et al., 2023).

For respiratory diseases, a marker-free SERS-based screening method utilizing a portable handheld Raman spectrophotometer is successfully developed to distinguish saliva samples between healthy individuals, COVID-19-infected and COVID-19-convalescent subjects (Figure 9B). Through a trained Support Vector Machine (SVM) classifier, authors obtain prediction accuracies of 95.00%, 94.73% and 95.28% for healthy individuals, COVID-19 infected patients and COVID-19 convalescents, respectively (Karunakaran et al., 2022). In lung cancer detection, studies showed saliva samples from patients could be analyzed through statistical SERS data analysis coupled with SVM and random forest algorithms, achieving 100% sensitivity and specificity in initial tests. The leave-one-out approach reached 95.08% sensitivity and 100% specificity, while the Random Forest method showed 96.72% sensitivity and 100% specificity (Qian et al., 2018). Advanced work developed three-dimensional plasmonic hexamer nanostructures (3D-PHP) coated on paper substrates for lung cancer detection using saliva samples (Figure 9C), achieving 91.2% sensitivity, 80.2% specificity and 87.5% accuracy, with AUC of 0.95 (Linh et al., 2024). For gastric cancer, investigations revealed specificity of 87.7% and sensitivity of 80.0% when differentiating between early/advanced patients and healthy individuals through marker-free saliva testing (Chen et al., 2018). An innovative approach implemented a machine-learning-based SERS spectral characterization algorithm for distinguishing exosomes from cancerous and non-cancerous sources (Figure 9D). Analysis of exosomes from tissues, blood and saliva showed prediction accuracies of 90.0%, 85.0% and 72.0%, with AUC values of 0.96, 0.91 and 0.65 respectively (Liu et al., 2022).

### 3.4 Detection of sweat and tear

**FIGURE 10:**
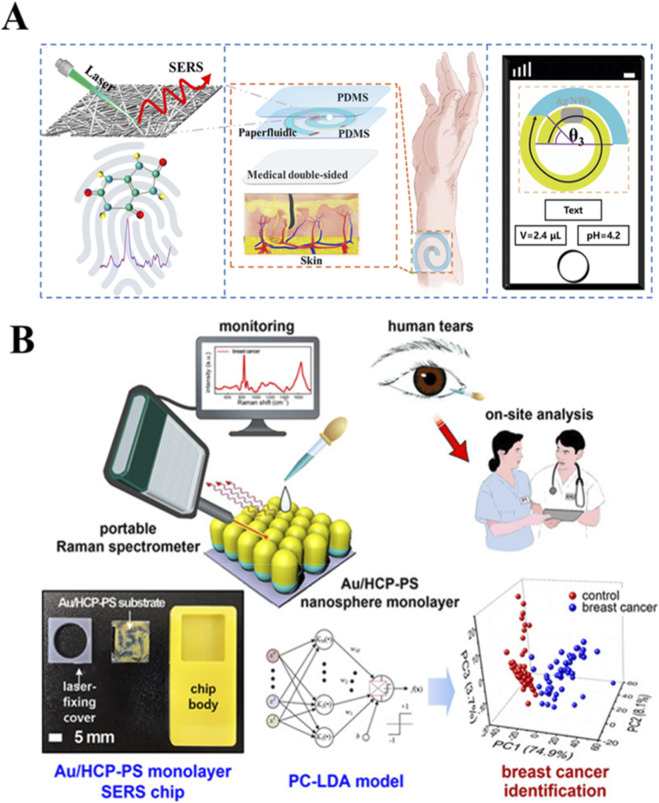
SERS-based approaches for label-free cancer detection using alternative biofluids **(A)** Wearable sweat sensor integrating SERS and AI for gout diagnosis (Chen et al., 2024). **(B)** Tears-based SERS biosensor for breast cancer detection (Kim et al., 2020).

Sweat has emerged as a promising biological fluid for SERS analysis due to its availability, non-invasiveness and painless collection. In a wearable platform designed for this purpose, a spiral channel comprising colorimetric paper embedded with Ag nanowires (AgNW) is used to capture sweat for subsequent SERS measurements. Leveraging smartphone imaging technology, pH levels and sweat volume can be accurately quantified through image recognition algorithms. For diagnosing gout, SERS spectra of human sweat containing uric acid are collected using this smart wearable platform. These SERS spectra are then analyzed using AI algorithms (Figure 10A). Results indicate that the ANN algorithm exhibits superior effectiveness in gout identification, achieving an accuracy of 97.0% (Chen et al., 2024).

Tear fluid, serving as a reservoir of biomarkers, presents distinct advantages over blood due to the absence of solid proteins, easy collection, non-invasiveness and patient comfort. Kim et al. utilized portable Raman spectroscopy along with a multivariate statistics-based identification algorithm to detect or predict asymptomatic breast cancer in human tears (Figure 10B). Employing a small sample size PC-LDA identification method, they achieved a classification accuracy of 96.0%. Notably, this method demonstrated clinical sensitivity of 92.0% and specificity of 100% for identifying asymptomatic breast cancer based on tear analysis (Kim et al., 2020).

## 4 Challenges and prospects

Recent advances in SERS-based liquid biopsy have demonstrated significant potential for transforming cancer diagnostics through label-free detection approaches. The fundamental principle of SERS detection relies on plasmonic enhancement at nanostructured surfaces, offering distinct advantages including single-molecule sensitivity and fingerprint-specific molecular identification while requiring minimal sample preparation. Substantial progress has been made in substrate development, with nanoparticle-based systems providing tunable plasmonic properties, two-dimensional substrates enabling uniform signal enhancement, and three-dimensional architectures creating high-density hot spots for improved detection limits.

Clinical applications of SERS have shown particularly promising results across multiple biofluids. Blood-based analyses have achieved diagnostic accuracy exceeding 90.0% for various cancer types, while urine assays demonstrate potential for noninvasive urological cancer screening. SERS profiling has emerged as a viable approach for both oral and systemic cancer detection. These technological advances collectively enable early cancer detection through sensitive biomarker identification and facilitate real-time treatment monitoring, representing a significant improvement over conventional invasive biopsy methods.

The field nevertheless faces several challenges that must be addressed for successful clinical translation. A fundamental and persistent challenge originates from the nanoscale heterogeneity of SERS substrates—specifically, the difficulty in fabricating uniform and stable distributions of plasmonic “hot spots.” This inherent variability directly undermines signal reproducibility and complicates reliable quantitative analysis, which are prerequisites for any clinical diagnostic tool. Consequently, translating the remarkable theoretical sensitivity of SERS into robust, standardized clinical assays remains a primary obstacle. Substantial hurdles remain to be overcome before the full clinical translation of label-free SERS liquid biopsy can be realized. A primary challenge lies in the lack of standardization across the entire workflow … Addressing these challenges of standardization, biological interpretation, and clinical validation is paramount for the future of the field. For example, standardization of sample processing protocols remains a critical hurdle, alongside the need for larger multicenter clinical validations. Further optimization of portable device designs will be essential for point-of-care implementation. While current results indicate SERS-based liquid biopsies could substantially reduce cancer mortality through earlier detection, additional research is needed to fully explore this potential and establish standardized clinical protocols. The integration of advanced machine learning algorithms with SERS detection platforms shows particular promise for overcoming current limitations in data interpretation and diagnostic accuracy.

## References

[B1] Albrecht M. G. Creighton J. A. (1977). Anomalously intense Raman spectra of pyridine at a silver electrode. J. Am. Chem. Soc., 99(15): 5215–5217. 10.1021/ja00457a071

[B2] Almeida D. A. L. Marques F. C. de Oliveira R. Barberes G. Peixoto L. P. F. Maia L. F. (2025). Raman spectroscopy integrated with Machine learning as a tool for maturity assessment of organic matter: a case study in santos basin, Brazil. ACS Earth Space Chem., 9(8): 2004–2016. 10.1021/acsearthspacechem.5c00068

[B3] Attwaters M. (2023). Cancer survival across continents. Nat. Cancer 4 (12), 1639. 10.1038/s43018-023-00665-1 38102353

[B4] Bai X. Lin J. Wu X. Lin Y. Zhao X. Du W. (2022). Label-free detection of bladder cancer and kidney cancer plasma based on SERS and multivariate statistical algorithm. Spectrochimica Acta Part A Mol. Biomol. Spectrosc. 279, 121336. 10.1016/j.saa.2022.121336 35605419

[B5] Batool S. M. Yekula A. Khanna P. Hsia T. Gamblin A. S. Ekanayake E. (2023). The liquid biopsy consortium: challenges and opportunities for early cancer detection and monitoring. Cell Rep. Med. 4 (10), 101198. 10.1016/j.xcrm.2023.101198 37716353 PMC10591039

[B6] Bi X. Lin L. Chen Z. Ye J. (2023). Artificial intelligence for surface-enhanced raman spectroscopy. Small Methods 8 (1), 2301243. 10.1002/smtd.202301243 37888799

[B7] Billimoria R. Bhatt P. (2023). Senescence in cancer: advances in detection and treatment modalities. Biochem. Pharmacol. 215, 115739. 10.1016/j.bcp.2023.115739 37562510

[B8] Borşa R.-M. Toma V. Onaciu A. Moldovan C. S. Mărginean R. Cenariu D. (2023). Developing new diagnostic tools based on SERS analysis of filtered salivary samples for oral cancer detection [J/OL]. Int. J. Mol. Sci. 24 (15), 12125. 10.3390/ijms241512125 37569501 PMC10418512

[B9] Cai C. Liu Y. Li J. Wang L. Zhang K. (2022). Serum fingerprinting by slippery liquid-infused porous SERS for non-invasive lung cancer detection. Analyst 147 (20), 4426–4432. 10.1039/D2AN01325H 36106390

[B10] Cao D. Lin H. Liu Z. Gu Y. Hua W. Cao X. (2022). Serum-based surface-enhanced Raman spectroscopy combined with PCA-RCKNCN for rapid and accurate identification of lung cancer. Anal. Chim. Acta 1236, 340574. 10.1016/j.aca.2022.340574 36396230

[B11] Cao D. Lin H. Liu Z. Qiu J. Ge S. Hua W. (2023). PCA-TLNN-based SERS analysis platform for label-free detection and identification of cisplatin-treated gastric cancer. Sensors Actuators B Chem. 375, 132903. 10.1016/j.snb.2022.132903

[B12] Cardinal M. F. Vander Ende E. Hackler R. A. McAnally M. O. Stair P. C. Schatz G. C. (2017). Expanding applications of SERS through versatile nanomaterials engineering. Chem. Soc. Rev. 46 (13), 3886–3903. 10.1039/c7cs00207f 28640313

[B13] ÇelİK Y. Kurt A. (2021). Three dimensional porous expanded graphite/silver nanoparticles nanocomposite platform as a SERS substrate. Appl. Surf. Sci. 568, 150946. 10.1016/j.apsusc.2021.150946

[B14] Chao L. Di X. Xuan D. Huang Q. (2022). A review: research progress of SERS-based sensors for agricultural applications. Trends Food Sci. and Technol. 128, 90–101. 10.1016/j.tifs.2022.07.012

[B15] Chen Y. Cheng S. Zhang A. Song J. Chang J. Wang K. (2018). Salivary analysis based on surface enhanced raman scattering sensors distinguishes early and advanced gastric cancer patients from healthy persons. J. Biomed. Nanotechnol. 14 (10), 1773–1784. 10.1166/jbn.2018.2621 30041723

[B16] Chen M. Liu D. Du X. Lo K. H. Wang S. Zhou B. (2020). 2D materials: excellent substrates for surface-enhanced Raman scattering (SERS) in chemical sensing and biosensing. Trends Anal. Chem. 130, 115983. 10.1016/j.trac.2020.115983

[B17] Chen W. Wang W. Xing H. Li W. He H. Li W. (2023a). Interfacial self-assembled dual-functional nanocomposite films for SERS monitoring of visible-light photocatalytic degradation of organic dye pollutants. Surfaces Interfaces 38, 102808. 10.1016/j.surfin.2023.102808

[B18] Chen S. Wu H. Chen C. Wang D. Yang Y. Zhou Z. (2023b). The prognostic prediction of periodontal non-surgery therapy in periodontitis patients based on surface-enhanced Raman measurements of pre-treatment saliva. Spectrochimica Acta Part A Mol. Biomol. Spectrosc. 288, 122150. 10.1016/j.saa.2022.122150 36459721

[B19] Chen Z. Wang W. Tian H. Yu W. Niu Y. Zheng X. (2024). Wearable intelligent sweat platform for SERS-AI diagnosis of gout. Lab a Chip 24 (7), 1996–2004. 10.1039/D3LC01094E 38373026

[B20] Cheng N. Fu J. Chen D. Chen S. Wang H. (2021a). An antibody-free liver cancer screening approach based on nanoplasmonics biosensing chips via spectrum-based deep learning. NanoImpact 21, 100296. 10.1016/j.impact.2021.100296 35559784

[B21] Cheng N. Chen D. Lou B. Fu J. Wang H. (2021b). A biosensing method for the direct serological detection of liver diseases by integrating a SERS-based sensor and a CNN classifier. Biosens. Bioelectron. 186, 113246. 10.1016/j.bios.2021.113246 33965791

[B22] Choi N. Dang H. Das A. Sim M. S. Chung I. Y. Choo J. (2020). SERS biosensors for ultrasensitive detection of multiple biomarkers expressed in cancer cells. Biosens. Bioelectron. 164, 112326. 10.1016/j.bios.2020.112326 32553352

[B23] Del Mistro G. Cervo S. Mansutti E. Spizzo R. Colombatti A. Belmonte P. (2015). Surface-enhanced Raman spectroscopy of urine for prostate cancer detection: a preliminary study. Anal. Bioanal. Chem. 407 (12), 3271–3275. 10.1007/s00216-015-8610-9 25791298

[B24] Diao X. Li X. Hou S. Li H. Qi G. Jin Y. (2023). Machine learning-based label-free SERS profiling of exosomes for accurate fuzzy diagnosis of cancer and dynamic monitoring of drug therapeutic processes. Anal. Chem. 95 (19), 7552–7559. 10.1021/acs.analchem.3c00026 37139959

[B25] Ding S.-Y. You E.-M. Tian Z.-Q. Moskovits M. (2017). Electromagnetic theories of surface-enhanced Raman spectroscopy. Chem. Soc. Rev. 46 (13), 4042–4076. 10.1039/C7CS00238F 28660954

[B26] Faur C. I. Dinu C. Toma V. Jurj A. Mărginean R. Onaciu A. (2023). A new detection method of oral and oropharyngeal squamous cell carcinoma based on multivariate analysis of surface enhanced Raman spectra of salivary exosomes [J/OL]. J. Pers. Med. 13 (5), 762DOI. 10.3390/jpm13050762 37240933 PMC10219614

[B27] Fleischmann M. Hendra P. J. McQuillan A. J. (1974). Raman spectra of pyridine adsorbed at a silver electrode. Chem. Phys. Lett. 26 (2), 163–166. 10.1016/0009-2614(74)85388-1

[B28] Fu M. Li Y. Wang J. (2025). Incidence and mortality of colorectal cancer in Asia in 2022 and projections for 2050. J. Gastroenterology Hepatology 40 (5), 1143–1156. 10.1111/jgh.16910 40018878

[B29] Gao N. Wang Q. Tang J. Yao S. Li H. Yue X. (2021). Non-invasive SERS serum detection technology combined with multivariate statistical algorithm for simultaneous screening of cervical cancer and breast cancer. Anal. Bioanal. Chem. 413 (19), 4775–4784. 10.1007/s00216-021-03431-3 34128082

[B30] Gao S. Lin Y. Zhao X. Gao J. Xie S. Gong W. (2022). Label-free surface enhanced Raman spectroscopy analysis of blood serum via coffee ring effect for accurate diagnosis of cancers. Spectrochimica Acta Part A Mol. Biomol. Spectrosc. 267, 120605. 10.1016/j.saa.2021.120605 34802933

[B31] Gao N. Wang Q. Tang J. Lv X. Li H. Yue X. (2023). Serum SERS spectroscopy combined with classification algorithm in the non-destructive identification of cervical cancer. Appl. Phys. A 129 (12), 822. 10.1007/s00339-023-07116-9

[B32] Ge H. Gao X. Lin J. Zhao X. Wu X. Zhang H. (2024). Label-free SERS detection of prostate cancer based on multi-layer perceptron surrogate model method. Spectrochimica Acta Part A Mol. Biomol. Spectrosc. 304, 123407. 10.1016/j.saa.2023.123407 37717486

[B33] Guselnikova O. Lim H. Kim H.-J. Kim S. H. Gorbunova A. Eguchi M. (2022). New trends in nanoarchitectured SERS substrates: nanospaces, 2D materials, and organic heterostructures. Small 18 (25), 2107182. 10.1002/smll.202107182 35570326

[B34] Han X. X. Rodriguez R. S. Haynes C. L. Ozaki Y. Zhao B. (2022). Surface-enhanced Raman spectroscopy. Nat. Rev. Methods Prim. 1 (1), 87. 10.1038/s43586-021-00083-6

[B35] Hu D. Xu X. Zhao Z. Li C. Tian Y. Liu Q. (2021). Detecting urine metabolites of bladder cancer by surface-enhanced Raman spectroscopy. Spectrochimica Acta Part A Mol. Biomol. Spectrosc. 247, 119108. 10.1016/j.saa.2020.119108 33161263

[B36] Hu Y. Xu L. Miao X. Xie Y. Zhang Z. Wang Y. (2025). SERS/Fluorescence dual-modal imaging bioprobe for accurate diagnosis of breast cancer. Anal. Chem. 97 (10), 5527–5537. 10.1021/acs.analchem.4c05833 40025760

[B37] Humaira K. Shah M. R. Barek J. Malik M. I. (2022). Cancer biomarkers and their biosensors: a comprehensive review. Trends Anal. Chem. 158, 116813. 10.1016/j.trac.2022.116813

[B38] Indrajit S. Ruiyang X. Jamie J. Rhee H. Flatt K. Gruev V. (2022). Biomimetic surface-enhanced raman scattering nanoparticles with improved dispersibility, signal brightness, and tumor targeting functions. ACS Nano 16 (5), 8051–8063. 10.1021/acsnano.2c01062 35471820

[B39] Itoh T. Yamamoto Y. S. Ozaki Y. (2017). Plasmon-enhanced spectroscopy of absorption and spontaneous emissions explained using cavity quantum optics. Chem. Soc. Rev. 46 (13), 3904–3921. 10.1039/C7CS00155J 28653715

[B40] Jia Y. Li Y. Bai X. Liu L. Shan Y. Wang F. (2025). Raman spectroscopy and exosome-based machine learning predicts the efficacy of neoadjuvant therapy for HER2-Positive breast cancer. Anal. Chem. 97 (2), 1374–1385. 10.1021/acs.analchem.4c05833 39780544

[B41] Jin J. Guo Z. Fan D. Zhao B. (2022). Spotting the driving forces for SERS of two-dimensional nanomaterials. Mater. Horizons 10 (4), 1087–1104. 10.1039/d2mh01241c 36629521

[B42] Jin J. Nadia M. Sang-Min P. Wu Y. L. Sample A. D. Diloknawarit B. (2023). Spike growth on patterned gold nanoparticle scaffolds. Nano Lett. 23 (23), 11260–11265. 10.1021/acs.nanolett.3c03778 38048438

[B43] Joseph M. M. Narayanan N. Nair J. B. Karunakaran V. Ramya A. N. Sujai P. T. (2018). Exploring the margins of SERS in practical domain: an emerging diagnostic modality for modern biomedical applications. Biomaterials 181, 140–181. 10.1016/j.biomaterials.2018.07.045 30081304

[B44] Karunakaran V. Joseph M. M. Yadev I. Sharma H. Shamna K. Saurav S. (2022). A non-invasive ultrasensitive diagnostic approach for COVID-19 infection using salivary label-free SERS fingerprinting and artificial intelligence. J. Photochem. Photobiol. B Biol. 234, 112545. 10.1016/j.jphotobiol.2022.112545 36049288 PMC9389522

[B45] Kim S. Kim T. G. Lee S. H. Kim W. Bang A. Moon S. W. (2020). Label-free surface-enhanced raman spectroscopy biosensor for On-Site breast cancer detection using human tears. ACS Appl. Mater. and Interfaces 12 (7), 7897–7904. 10.1021/acsami.9b19421 31971765

[B46] Kim W. H. Lee J. U. Jeon M. J. Park K. H. Sim S. J. (2022). Three-dimensional hierarchical plasmonic nano-architecture based label-free surface-enhanced Raman spectroscopy detection of urinary exosomal miRNA for clinical diagnosis of prostate cancer. Biosens. Bioelectron. 205, 114116. 10.1016/j.bios.2022.114116 35235898

[B47] Langer J. Jimenez de Aberasturi D. Aizpurua J. Alvarez-Puebla R. A. Auguié B. Baumberg J. J. (2020). Present and future of surface-enhanced raman scattering. ACS Nano 14 (1), 28–117. 10.1021/acsnano.9b04224 31478375 PMC6990571

[B48] Lee S. Jue M. Lee K. Paulson B. Oh J. Cho M. (2024). Early-stage diagnosis of bladder cancer using surface-enhanced Raman spectroscopy combined with machine learning algorithms in a rat model. Biosens. Bioelectron. 246, 115915. 10.1016/j.bios.2023.115915 38081101

[B49] Lei X. Yujiao X. Jie L. Wu A. Jiang T. (2023). Advancements in SERS-based biological detection and its application and perspectives in pancreatic cancer. VIEW 5 (1), 20230070. 10.1002/viw.20230070

[B50] Li R. Gui B. Mao H. Yang Y. Chen D. Xiong J. (2020). Self-concentrated surface-enhanced raman scattering-active droplet sensor with three-dimensional hot spots for highly sensitive molecular detection in complex liquid environments. ACS Sensors 5 (11), 3420–3431. 10.1021/acssensors.0c01276 32929960

[B51] Li J. Li W. Rao Y. Shi F. Yu S. Yang H. (2021). Synthesis of highly ordered AgNPs-coated silica photonic crystal beads for sensitive and reproducible 3D SERS substrates. Chin. Chem. Lett., 32(1): 150–153. 10.1016/j.cclet.2020.10.043

[B52] Li H. Zhang S. Zhu R. Zhou Z. Xia L. Lin H. (2022). Early assessment of chemotherapeutic response in hepatocellular carcinoma based on serum surface-enhanced raman spectroscopy. Spectrochimica Acta Part A Mol. Biomol. Spectrosc. 278, 121314. 10.1016/j.saa.2022.121314 35525180

[B53] Li D. Ju F. Wang H. Fan C. Jacob J. C. Gul S. (2023a). Combination of the biomarkers for aging and cancer? - challenges and current status. Transl. Oncol. 38, 101783. 10.1016/j.tranon.2023.101783 37716258 PMC10514562

[B54] Li Q. Huo H. Wu Y. Chen L. Su L. Zhang X. (2023b). Design and synthesis of SERS materials for *in vivo* molecular imaging and biosensing. Adv. Sci. 10 (8), 2202051. 10.1002/advs.202202051 36683237 PMC10015885

[B55] Li J. She Q. Wang W. Liu R. You R. Wu Y. (2023c). Label-free SERS analysis of serum using Ag NPs/Cellulose nanocrystal/graphene oxide nanocomposite film substrate in screening Colon cancer [J/OL]. Nanomater. (Basel). 13 (2), 334. 10.3390/nano13020334 36678088 PMC9864651

[B56] Li J. Yang L. Shi F. Long Y. Wang Y. Stuart D. D. (2025). Versatile Au nanozyme Raman probe strategy for ultrasensitive encoded photonic crystal-based SERS multiplex immunosensing. Chin. Chem. Lett., 36: 110883. 10.1016/j.cclet.2025.110883

[B57] Liang X. Miao X. Xiao W. Ye Q. Wang S. Lin J. (2020). Filter-membrane-based ultrafiltration coupled with surface-enhanced raman spectroscopy for potential differentiation of benign and malignant thyroid tumors from blood plasma. Int. J. Nanomedicine 15, 2303–2314. 10.2147/ijn.s233663 32280222 PMC7132009

[B58] Lie J. Luo F. Liu Y. Yang Y. Nie Q. Chen X. (2024). Recyclable magnetic nanoparticles combined with TiO2 enrichment and “Off” to “On” SERS assay for sensitive detection of alkaline phosphatase. Chem. Eng. J. 479, 147241. 10.1016/j.cej.2023.147241

[B59] Liebel M. Calderon I. Pazos-Perez N. van Hulst N. F. Alvarez-Puebla R. A. (2022). Widefield SERS for high-throughput nanoparticle screening. Angew. Chem. Int. Ed. 61 (20), e202200072. 10.1002/anie.202200072 35107845

[B60] Lin X. Lin D. Chen Y. Lin J. Weng S. Song J. (2021a). High throughput blood analysis based on deep learning algorithm and self-positioning super-hydrophobic SERS platform for non-invasive multi-disease screening. Adv. Funct. Mater. 31 (51), 2103382. 10.1002/adfm.202103382

[B61] Lin Y. Gao J. Tang S. Zhao X. Zheng M. Gong W. (2021b). Label-free diagnosis of breast cancer based on serum protein purification assisted surface-enhanced Raman spectroscopy. Spectrochimica Acta Part A Mol. Biomol. Spectrosc. 263, 120234. 10.1016/j.saa.2021.120234 34343842

[B62] Linh V. T. N. Lee M.-Y. Mun J. Kim Y. Kim H. Han I. W. (2023). 3D plasmonic coral nanoarchitecture paper for label-free human urine sensing and deep learning-assisted cancer screening. Biosens. Bioelectron. 224, 115076. 10.1016/j.bios.2023.115076 36641876

[B63] Linh V. T. N. Kim H. Lee M.-Y. Mun J. Kim Y. Jeong B. H. (2024). 3D plasmonic hexaplex paper sensor for label-free human saliva sensing and machine learning-assisted early-stage lung cancer screening. Biosens. Bioelectron. 244, 115779. 10.1016/j.bios.2023.115779 37922808

[B87] Liu R. Yan X. Zhang B. Chen Y. Liu Y. Lu Y. (2023). Ag nanocubes/cationic cellulose nanofibers/polyacrylamide hydrogel as a SERS platform for *in situ* separation, rapid enrichment and sensitive detection of anticancer drugs in plasma. Sensors Actuators B Chem. 402, 135126. 10.1016/j.snb.2023.135126

[B64] Liu Z. Li T. Wang Z. Liu J. Huang S. Min B. H. (2022). Gold nanopyramid arrays for non-invasive surface-enhanced raman spectroscopy-based gastric cancer detection via sEVs. ACS Appl. Nano Mater. 5 (9), 12506–12517. 10.1021/acsanm.2c01986 36185166 PMC9513748

[B65] Long Y. Li J. Shi F. Xue Y. Wang Y. Yang Z. (2025). New-typed Co_3_O_4_@Co–Fe Oxide/AuNPs double nanoboxes nanozyme enables bimodal monitoring metallothioneins. Anal. Chem., 97(40): 22279–22287. 10.1021/acs.analchem.5c04519 41036969

[B67] Lu L. Sun M. Lu Q. Wu T. Huang B. (2021). High energy X-ray radiation sensitive scintillating materials for medical imaging, cancer diagnosis and therapy. Nano Energy 79, 105437. 10.1016/j.nanoen.2020.105437

[B68] Lu D. Huang Z. Chen J. Lu Y. (2025). pH-Adjusted liquid SERS approach: toward a reliable plasma-based early stage lung cancer detection. Anal. Chem. 97 (1), 508–515. 10.1021/acs.analchem.4c04671 39723763

[B69] Lussier F. Thibault V. Charron B. Wallace G. Q. Masson J. F. (2020). Deep learning and artificial intelligence methods for raman and surface-enhanced Raman scattering. Trends Anal. Chem. 124, 115796. 10.1016/j.trac.2019.115796

[B70] Ma Y. Chi J. Zheng Z. Attygalle A. Kim I. Y. Du H. (2021). Therapeutic prognosis of prostate cancer using surface-enhanced Raman scattering of patient urine and multivariate statistical analysis. J. Biophot. 14 (1), e202000275. 10.1002/jbio.202000275 32909380

[B71] Moisoiu V. Socaciu A. Stefancu A. Iancu S. D. Boros I. Alecsa C. D. (2019). Breast cancer diagnosis by surface-enhanced raman scattering (SERS) of urine [J/OL]. Appl. Sci. (Basel). 9 (4), 806DOI. 10.3390/app9040806

[B72] Moutinho S. (2023). Clinical trials assess a precision-medicine approach to cancer screening. Nat. Med. 29 (7), 1587–1590. 10.1038/s41591-023-02431-3 37464042

[B73] Nargis H. F. Nawaz H. Bhatti H. N. Jilani K. Saleem M. (2020). Comparison of surface enhanced Raman spectroscopy and Raman spectroscopy for the detection of breast cancer based on serum samples. Spectrochimica Acta Part A Mol. Biomol. Spectrosc. 246, 119034. 10.1016/j.saa.2020.119034 33049470

[B74] Nguyen T. M. Jeong S. S. Kang S. K. Han S.-W. Nguyen T. M. T. Lee S. (2024). 3D superclusters with hybrid bioinks for early detection in breast cancer. ACS Sensors 9 (2), 699–707. 10.1021/acssensors.3c01938 38294962 PMC10897927

[B75] Panikar S. S. Sekhar Reddy K. C. Gonzalez A. L. Ramírez-García G. Rodríguez Á. G. Mondragon Sosa M. A. (2022). Deep eutectic solvent-enabled plasmonic nanocellulose aerogel: on-demand three-dimensional (3D) SERS hotspot based on collapsing mechanism. Anal. Chem. 94 (47), 16470–16480. 10.1021/acs.analchem.2c03964 36318661

[B76] Park J. Hwang M. Choi B. Jeong H. Jung J. H. Kim H. K. (2017). Exosome classification by pattern analysis of surface-enhanced raman spectroscopy data for lung cancer diagnosis. Anal. Chem. 89 (12), 6695–6701. 10.1021/acs.analchem.7b00911 28541032

[B77] Paz M. M. Veiga A. P. Regueira T. Vázquez C. V. Arturo López Quintela M. (2023). Facile generation of surface diversity in gold nanoparticles. J. Colloid Interface Sci. 641, 719–728. 10.1016/j.jcis.2023.03.043 36972622

[B78] Peng S. Lu D. Zhang B. You R. Chen J. Xu H. (2023). Machine learning–assisted internal standard calibration label-free SERS strategy for colon cancer detection. Anal. Bioanal. Chem. 415 (9), 1699–1707. 10.1007/s00216-023-04566-1 36781448

[B79] Phan-Quang G. C. Han X. Koh C. S. L. Sim H. Y. F. Lay C. L. Leong S. X. (2019). Three-dimensional surface-enhanced raman scattering platforms: large-scale plasmonic hotspots for new applications in sensing, microreaction, and data storage. Accounts Chem. Res. 52 (7), 1844–1854. 10.1021/acs.accounts.9b00163 31180637

[B80] Phyo J. B. Woo A. Yu H. J. Lim K. Cho B. H. Jung H. S. (2021). Label-free SERS analysis of urine using a 3D-Stacked AgNW-Glass fiber filter sensor for the diagnosis of pancreatic cancer and prostate cancer. Anal. Chem. 93 (8), 3778–3785. 10.1021/acs.analchem.0c04200 33576598

[B81] Pickhardt P. J. (2022). Value-added opportunistic CT screening: state of the art. Radiology 303 (2), 241–254. 10.1148/radiol.211561 35289661 PMC9083232

[B82] Qi Y. Brasiliense V. Ueltschi T. W. Park J. E. Wasielewski M. R. Schatz G. C. (2020). Plasmon-driven chemistry in Ferri-/Ferrocyanide gold nanoparticle oligomers: a SERS study. J. Am. Chem. Soc. 142 (30), 13120–13129. 10.1021/jacs.0c05031 32618467

[B83] Qian K. Wang Y. Hua L. Chen A. Zhang Y. (2018). New method of lung cancer detection by saliva test using surface-enhanced Raman spectroscopy. Thorac. Cancer 9 (11), 1556–1561. 10.1111/1759-7714.12837 30168669 PMC6209779

[B84] Qian H. Y. Shao X. G. Zhang H. Wang Y. Liu S. Pan J. (2022). Diagnosis of urogenital cancer combining deep learning algorithms and surface-enhanced Raman spectroscopy based on small extracellular vesicles. Spectrochim. Acta A 281, 121603. 10.1016/j.saa.2022.121603 35868057

[B85] Raman C. V. Krishnan K. S. (1928). A new type of secondary radiation. Nature 121 (3048), 501–502. 10.1038/121501c0

[B86] Ramos D. Malvar O. Davis Z. J. Tamayo J. Calleja M. (2018). Nanomechanical plasmon spectroscopy of single gold nanoparticles. Nano Lett. 18 (11), 7165–7170. 10.1021/acs.nanolett.8b03236 30339403

[B88] Shao X. Zhang H. Wang Y. Qian H. Zhu Y. Dong B. (2020). Deep convolutional neural networks combine Raman spectral signature of serum for prostate cancer bone metastases screening. Nanomedicine Nanotechnol. Biol. Med. 29, 102245. 10.1016/j.nano.2020.102245 32592757

[B89] She Q. Li J. Lu Y. You R. (2021). *In situ* synthesis of silver nanoparticles on dialdehyde cellulose as reliable SERS substrate. Cellulose 28 (17), 10827–10840. 10.1007/s10570-021-04224-8

[B90] Shin H. Oh S. Hong S. Kang M. Kang D. Ji Y. G. (2020). Early-stage lung cancer diagnosis by deep learning-based spectroscopic analysis of circulating exosomes. ACS Nano 14 (5), 5435–5444. 10.1021/acsnano.9b09119 32286793

[B91] Shin H. Choi B. H. Shim O. Kim J. Park Y. Cho S. K. (2023). Single test-based diagnosis of multiple cancer types using Exosome-SERS-AI for early stage cancers. Nat. Commun. 14 (1), 1644. 10.1038/s41467-023-37403-1 36964142 PMC10039041

[B92] Shuyan R. Wenxi W. Liting Q. Yan X. Peng Z. Zhu H. (2024). Preparation of 3D flexible SERS substrates by mixing gold nanorods in hydrogels for the detection of malachite green and crystal violet. Microchim. Acta 191 (4), 205. 10.1007/s00604-024-06284-6 38492087

[B93] Sofie Van D. Philippe T. Hannelore D. Hendrix A. (2024). Towards the clinical implementation of extracellular vesicle-based biomarker assays for cancer. Clin. Chem. 70 (1), 165–178. 10.1093/clinchem/hvad189 38175582

[B94] Son J. Kim G.-H. Lee Y. Lee C. Cha S. Nam J. M. (2022). Toward quantitative surface-enhanced raman scattering with plasmonic nanoparticles: multiscale view on heterogeneities in particle morphology, surface modification, interface, and analytical protocols. J. Am. Chem. Soc. 144 (49), 22337–22351. 10.1021/jacs.2c05950 36473154

[B95] Sun Y. Zhou L. Ding Y. Liu C. Mao Z. S. Jiang Q. Y. (2024). Fabrication of flexible electrospinning nano-fiber membrane for detection of respiratory tract transmission virus based on SERS. Talanta 266, 125127. 10.1016/j.talanta.2023.125127 37647815

[B96] Vargas-Obieta E. Martínez-Espinosa J. C. Martínez-Zerega B. E. Jave-Suárez L. F. Aguilar-Lemarroy A. González-Solís J. L. (2016). Breast cancer detection based on serum sample surface enhanced Raman spectroscopy. Lasers Med. Sci. 31 (7), 1317–1324. 10.1007/s10103-016-1976-x 27289243

[B66] Vázquez-Iglesias L. Casagrande G. M. S. García-Lojo D. Leal L. F. Ngo T. A. Pérez-Juste J. (2023). SERS sensing for cancer biomarker: approaches and directions. Bioact. Mater. 34, 248–268. 10.1016/j.bioactmat.2023.12.018 38260819 PMC10801148

[B97] Villazon J. Dela Cruz N. Shi L. (2025). Cancer cell line classification using raman spectroscopy of cancer-derived exosomes and machine learning. Anal. Chem. 97 (13), 7289–7298. 10.1021/acs.analchem.4c06966 40145503 PMC11983372

[B98] Wang Z. Zong S. Wu L. Zhu D. Cui Y. (2017). SERS-activated platforms for immunoassay: probes, encoding methods, and applications. Chem. Rev. 117 (12), 7910–7963. 10.1021/acs.chemrev.7b00027 28534612

[B99] Wang L. Wang X. Cheng L. Ding S. Wang G. Choo J. (2021). SERS-based test strips: principles, designs and applications. Biosens. Bioelectron. 189, 113360. 10.1016/j.bios.2021.113360 34051383

[B100] Wang W. Ruan S. Su Z. Xu P. Chen Y. Lin Z. (2023a). A novel “on–off” SERS nanoprobe based on sulfonated cellulose nanofiber-Ag composite for selective determination of NADH in human serum. Microchim. Acta 190 (7), 254. 10.1007/s00604-023-05809-9 37294367

[B101] Wang Y. Qian H. Shao X. Zhang H. Liu S. Pan J. (2023b). Multimodal convolutional neural networks based on the Raman spectra of serum and clinical features for the early diagnosis of prostate cancer. Spectrochimica Acta Part A Mol. Biomol. Spectrosc. 293, 122426. 10.1016/j.saa.2023.122426 36787677

[B102] Welch H. G. Bergmark R. (2024). Cancer screening, incidental detection, and overdiagnosis. Clin. Chem. 70 (1), 179–189. 10.1093/clinchem/hvad127 37757858

[B103] Xia L. Lu J. Chen Z. Cui X. Chen S. Pei D. (2021). Identifying benign and malignant thyroid nodules based on blood serum surface-enhanced Raman spectroscopy. Nanomedicine Nanotechnol. Biol. Med. 32, 102328. 10.1016/j.nano.2020.102328 33181274

[B104] Xia K. Yamaguchi K. Suzuki K. (2022). Recent advances in hybrid materials of metal nanoparticles and polyoxometalates. Angew. Chem. Int. Ed. 62 (1), e202214506. 10.1002/anie.202214506 36282183

[B105] Xiao T. Qi H. Xiangyu H. Lan L. Li M. Yao L. (2024). Exploring and engineering 2D transition metal dichalcogenides toward ultimate SERS performance. Adv. Mater. 36 (19), 2312348. 10.1002/adma.202312348 38302855

[B106] Xie Y. Su X. Wen Y. Zheng C. Li M. (2022). Artificial intelligent label-free SERS profiling of serum exosomes for breast cancer diagnosis and postoperative assessment. Nano Lett. 22 (19), 7910–7918. 10.1021/acs.nanolett.2c02928 36149810

[B107] Xu Y. Zhang Y. Li C. Ye Z. Bell S. E. J. (2023). SERS as a probe of surface chemistry enabled by surface-accessible plasmonic nanomaterials. Accounts Chem. Res. 56 (15), 2072–2083. 10.1021/acs.accounts.3c00207 37436068 PMC10399198

[B108] Xu J. Wang J. Zhang C. Zhao X. Yu J. Man B. (2024). Flexible multiscale cavity with omnidirectionality and high stability for in-site SERS detection of nanoplastics on oyster. Sensors Actuators B Chem. 403, 135218. 10.1016/j.snb.2023.135218

[B109] Yamamoto Y. S. Ozaki Y. Itoh T. (2014). Recent progress and frontiers in the electromagnetic mechanism of surface-enhanced Raman scattering. J. Photochem. Photobiol. C Photochem. Rev. 21, 81–104. 10.1016/j.jphotochemrev.2014.10.001

[B110] Yang W. Xia B. Wang L. Ma S. Liang H. Wang D. (2021). Shape effects of gold nanoparticles in photothermal cancer therapy. Mater. Today Sustain. 13, 100078. 10.1016/j.mtsust.2021.100078

[B111] Yang Y. Gao X. Zhang H. Chao F. Jiang H. Huang J. (2024). Multi-scale representation of surface-enhanced Raman spectroscopy data for deep learning-based liver cancer detection. Spectrochimica Acta Part A Mol. Biomol. Spectrosc. 308, 123764. 10.1016/j.saa.2023.123764 38134653

[B112] Yin Z. Xu K. Jiang S. Luo D. Chen R. Xu C. (2021). Recent progress on two-dimensional layered materials for surface enhanced Raman spectroscopy and their applications. Mater. Today Phys. 18, 100378. 10.1016/j.mtphys.2021.100378

[B113] Yin L. Fan M. She Q. You R. Lu Y. Lu D. (2022). Facilely self-assembled and dual-molecule calibration aptasensor based on SERS for ultra-sensitive detection of tetrodotoxin in pufferfish. Spectrochimica Acta Part A Mol. Biomol. Spectrosc. 279, 121275. 10.1016/j.saa.2022.121275 35605417

[B114] Yoshida K.-i. Itoh T. Tamaru H. Biju V. Ishikawa M. Ozaki Y. (2010). Quantitative evaluation of electromagnetic enhancement in surface-enhanced resonance Raman scattering from plasmonic properties and morphologies of individual Ag nanostructures. Phys. Rev. B 81 (11), 115406. 10.1103/PhysRevB.81.115406

[B115] You R. Y. Li J. Wang H. A. Wu Y. Weng J. Lu Y. (2023). High-performance SERS biosensor based on *in-situ* reduction of silver nanoparticles in an ultra-filtration centrifuge device for label-free detection of colon cancer in serum. J. Membr. Sci. 678, 121688. 10.1016/j.memsci.2023.121688

[B116] Zhang C. h. Cheng Y. Zhang S. Fan J. Gao Q. (2022). Changing epidemiology of hepatocellular carcinoma in Asia. Liver Int. 42 (9), 2029–2041. 10.1111/liv.15251 35319165

[B117] Zhang B. Nie Q. Yan X. Jiang Q. Ren J. Xu P. (2024). Machine learning-based SERS label-free detection of plasma and exosome binding in early-stage lung cancer. Microchem. J. 205, 111306. 10.1016/j.microc.2024.111306

[B118] Zhang C. Xu L. Miao X. Zhang D. Xie Y. Hu Y. (2025). Machine learning assisted dual-modal SERS detection for circulating tumor cells. Biosens. Bioelectron. 268, 116897. 10.1016/j.bios.2024.116897 39488132

